# Genomics Score Based on Genome-Wide Network Analysis for Prediction of Survival in Gastric Cancer: A Novel Prognostic Signature

**DOI:** 10.3389/fgene.2020.00835

**Published:** 2020-08-06

**Authors:** Zepang Sun, Hao Chen, Zhen Han, Weicai Huang, Yanfeng Hu, Mingli Zhao, Tian Lin, Jiang Yu, Hao Liu, Yuming Jiang, Guoxin Li

**Affiliations:** Department of General Surgery, Nanfang Hospital, Southern Medical University, Guangzhou, China

**Keywords:** gastric cancer, genome-wide network, miRNA, mRNA, DNA methylation, nomogram

## Abstract

**Purpose:**

Gastric cancer (GC) is a product of multiple genetic abnormalities, including genetic and epigenetic modifications. This study aimed to integrate various biomolecules, such as miRNAs, mRNA, and DNA methylation, into a genome-wide network and develop a nomogram for predicting the overall survival (OS) of GC.

**Materials and Methods:**

A total of 329 GC cases, as a training cohort with a random of 150 examples included as a validation cohort, were screened from The Cancer Genome Atlas database. A genome-wide network was constructed based on a combination of univariate Cox regression and least absolute shrinkage and selection operator analyses, and a nomogram was established to predict 1-, 3-, and 5-year OS in the training cohort. The nomogram was then assessed in terms of calibration, discrimination, and clinical usefulness in the validation cohort. Afterward, in order to confirm the superiority of the whole gene network model and further reduce the biomarkers for the improvement of clinical usefulness, we also constructed eight other models according to the different combinations of miRNAs, mRNA, and DNA methylation sites and made corresponding comparisons. Finally, Gene Ontology (GO), and Kyoto Encyclopedia of Genes and Genomes (KEGG) analyses were also performed to describe the function of this genome-wide network.

**Results:**

A multivariate analysis revealed a novel prognostic factor, a genomics score (GS) comprising seven miRNAs, eight mRNA, and 19 DNA methylation sites. In the validation cohort, comparing to patients with low GS, high-GS patients (HR, 12.886; *P* < 0.001) were significantly associated with increased all-cause mortality. Furthermore, after stratification of the TNM stage (I, II, III, and IV), there were significant differences revealed in the survival rates between the high-GS and low-GS groups as well (*P* < 0.001). The 1-, 3-, and 5-year C-index of whole genomics-based nomogram were 0.868, 0.895, and 0.928, respectively. The other models have comparable or relatively poor comprehensive performance, while they had fewer biomarkers. Besides that, DAVID 6.8 further revealed multiple molecules and pathways related to the genome-wide network, such as cytomembranes, cell cycle, and adipocytokine signaling.

**Conclusion:**

We successfully developed a GS based on genome-wide network, which may represent a novel prognostic factor for GC. A combination of GS and TNM staging provides additional precision in stratifying patients with different OS prognoses, constituting a more comprehensive sub-typing system. This could potentially play an important role in future clinical practice.

## Background

Gastric cancer (GC) is one of the most common malignant human tumors and the third leading cause of cancer-related mortalities worldwide. Reports estimate that nearly one million new cases and 800,000 deaths occur each year across the world (Torre et al., 2015). Despite the rapid research advancement, GC-related impacts on human life remain high around the globe. According to the global cancer burden data, hundreds of billions of dollars in economic losses are incurred each year due to GC. At the same time, stomach cancer has been reported to cause 19.1 million disability-adjusted life years, with 98% of these resulting from years of life lost and 2% from years lived with disability (Global Burden of Disease Cancer Collaboration et al., 2019).

Despite major breakthroughs in GC prevention, diagnosis, and treatment therapies reported over the past decade, prognosis remains a challenge at different TNM stages (Jiang et al., 2018a; Sun et al., 2019a,b). Notably, patients with similar clinical features and at the same tumor stage who receive uniform treatment have exhibited varying clinical outcomes (Bang et al., 2012; Jiang et al., 2018b). Such evidence indicates the existing challenges to traditional TNM staging (Serra et al., 2019), possibly due to a lack of molecular tools for effectively predicting the prognosis and the therapeutic effect of GC patients. Therefore, more rigorous and reliable systems that accurately reflect the heterogeneity of different patients and guide the development of treatment approaches are urgently needed (Duarte et al., 2018; Serra et al., 2019).

Tumors are a product of multiple genetic mutations, including genetic (gene expression) and epigenetic (DNA methylation and histone modification) modifications, as well as deregulations of tumor-suppressor genes and proto-oncogenes (Anna et al., 2018; Choi et al., 2019). In addition, changes in a set of genetic materials have been closely associated with cancer outcomes (Anna et al., 2018; Choi et al., 2019). Therefore, to effectively predict the prognosis of tumors, such as GC, a single biomarker is insufficient, necessitating the need for a gene network.

A variety of mRNAs have been associated with GC prognosis (Camargo et al., 2019), with microRNAs (miRNAs) also implicated in tumor prediction in the recent years (Li et al., 2010; Ueda et al., 2010; Camargo et al., 2019). These small, non-coding RNAs, comprising 22 nucleotides, primarily function to regulate protein translation by inhibiting the expression of target messenger RNAs (mRNAs). Apart from genetics, epigenetics is currently receiving considerable research attention. DNA methylation is the most common epigenetic event associated with cancer development and progression (Anna et al., 2018). Consequently, numerous studies have implicated DNA methylation in the diagnosis and the prognosis of various tumors, including GC (Camargo et al., 2019; Choi et al., 2019). Although these studies have revealed several biomarkers that have proved to be prognostic predictors in GC, only a handful have been adopted in clinical therapies or are used to build predictive models for the disease (Anna et al., 2018; Duarte et al., 2018; Camargo et al., 2019; Choi et al., 2019; Serra et al., 2019).

Previous studies have identified and recommended numerous biomarkers for GC. However, since malignant tumors often involve multiple layers and different levels of genetic changes, including the genome, transcriptome, and proteome, or even epigenetic content, selecting reasonable candidate factors from tens of thousands of biomarkers and comprehensively analyzing them as an independent feature is imperative to effectively develop a suitable prognostic target. Therefore, genetic networks containing a panel of abnormal factors from different regulatory levels represent the best chance for achieving prognostic value.

The whole genome-wide network analysis is reported in several other cancers, such as colorectal cancer, breast cancer, and lung cancer (Hou et al., 2018; Zhang et al., 2018), and it shows great value in differentiating the prognosis of these patients. Therefore, it is feasible and advantageous to apply genome-wide network analysis to GC.

In the current study, we performed a series of sophisticated statistical analyses and identified 33 genetic molecules that were highly correlated with the prognosis of GC. Specifically, we screened The Cancer Genome Atlas (TCGA), a genome project with 33 types of cancer, including gene expression, and DNA methylation as well as other biological information. Furthermore, we extended these independent prognostic factors to the “omics” concept. Then, a genome-wide network was constructed. Interestingly, the genomics score (GS) obtained herein could supplement TNM staging and enhance the prognostic value of different patients. Moreover, we developed multiple prognostic models, then validated, and compared them to ascertain their strengths and weaknesses. Finally, we performed pathway enrichment and gene oncology annotation analyses to elucidate the function of this gene network.

## Materials and Methods

### Data Acquisition and Preprocessing

**FIGURE 1:**
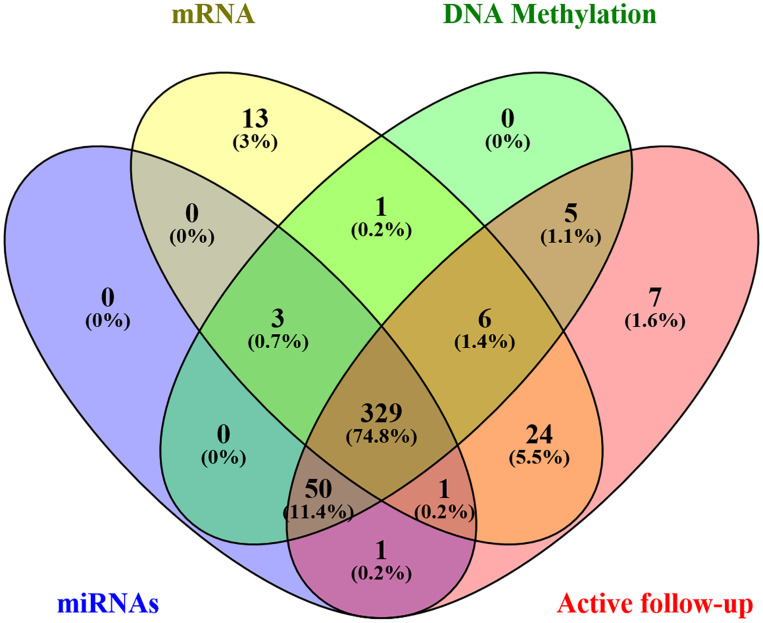
A Venn diagram displays the patients’ screening process.

Level 3 data were downloaded from the TCGA database using TCGA-Assembler Module A, in January 2019, which was then pretreated with Module B. The dataset comprised of clinical variables from 443 patients, including age, sex, stage, primary site, grade, treatment, and survival, as well as associated genome-wide data. In addition, the expression levels of 1,871 miRNAs, 20,531 mRNA, and 485,577 DNA methylation sites (Illumina methylation 450) were obtained from 384, 377, and 394 patients, respectively. Afterward, an intersection with a total of 332 samples was eventually retained. Furthermore, patients with missing active follow-up data were excluded from the analysis, leaving 329 patients in the final cohort (Figure 1). Moreover, genome-wide level 3 data whose expression levels for miRNAs, mRNA, and DNA methylation sites were missing in more than 50% of all samples were excluded from the final analysis. Finally, 329 GC patients with 566 miRNAs, 17,963 mRNA, and 396,081 DNA methylation sites were chosen for further analysis.

### Genome-Wide Network Analysis

Gene expression and DNA methylation data were normalized using R package before subsequent processing. Univariate and least absolute shrinkage and selection operator (LASSO) Cox regression models were combined and used to identify the most useful prognostic factors in miRNAs, mRNA, and DNA methylation sites associated with survival. Firstly, univariate Cox regression was performed on each candidate miRNA, mRNA, and DNA methylation site to elucidate its role in patient survival, then signatures with *P* value less than 0.05 were retained for subsequent analysis. Thereafter, the LASSO Cox regression model was applied to select and shrink the data (Supplementary Figure S3; Tibshirani, 1997). Finally, a GS, based on a genome-wide network comprising seven miRNAs, eight mRNAs, and 19 DNA methylation sites, was constructed for predicting survival. A summary of the whole screening process is displayed in Supplementary Figure S1.

### Development and Comparison of Individualized Prediction Models

The TCGA cohort with 329 cases was used as the training set, with a random 150 cases from the total cohort included as a validation group. The random number is 1,356. Firstly, we developed the original GS based on 34 biomarkers (seven miRNAs, eight mRNAs, and 19 DNA methylation sites). Then, considering the complexity of the original GS and difficult clinical application, in order to obtain a more concise and effective GS, we also constructed eight other models according to the different combinations of miRNAs, mRNA, and DNA methylation and made corresponding comparisons. Finally, a total of nine GS models based on the genome-wide network from LASSO were adopted to screen for the most appropriate markers. These included the following models: genomics (seven miRNAs, eight mRNA, and 19 DNA methylation sites), miRNAs (seven miRNAs), mRNA (eight mRNA), methylation (19 DNA methylation sites), miRNAs + methylation (seven miRNAs and 19 DNA methylation sites), miRNAs + mRNA (seven miRNAs and eight mRNA), mRNA + methylation (eight mRNA and 19 DNA methylation sites), Cox-model 1 (two miRNAs, six mRNA, and nine DNA methylation sites), and Cox-model 2 (one miRNA, one mRNA, and seven DNA methylation sites). Among them, markers from Cox-model 1 were separately detected from miRNAs, mRNA, or DNA methylation sites using multivariate Cox regression analysis after LASSO (Supplementary Tables S2–S4). On the other hand, markers from Cox-model 2 depended on signatures from a multivariate Cox regression analysis combining the genome-wide network and the clinical characteristics (Supplementary Table S5). Thereafter, we constructed several nomograms by incorporating significant (*P* < 0.05) GS variables and other clinical features following multivariate Cox regression (Iasonos et al., 2008), and a clinical nomogram was built as a blank control. The equations used for calculating the GS of these models are listed in Supplementary Table S6.

To calculate the discrimination and the stability of different Cox regression models, we applied C-statistics and calibration. Additionally, we performed an analysis of time-dependent receiver operator characteristics (ROC), based on the 1-, 3-, and 5-year survival endpoints, to assess the prognostic accuracy of the different nomograms. Furthermore, we evaluated the potential net benefit of different predictive models using decision curve analysis (DCA). DCA compares the clinical usefulness of different indicators by calculating the potential net benefit of each decision strategy at each threshold probability. Thus, DCA was a significant novel approach for comparing the old and the new models (Vickers and Elkin, 2006).

### Screening for Potential miRNA Target Genes

We predicted the potential target genes of the seven miRNAs, from LASSO, by screening the miRTarBase, miRDB, and TargetScan databases. Common genes from each miRNA across the three databases were then used for subsequent studies. More than 90% of the miRNAs showed negative regulation to target genes. Consequently, the expression data from TCGA were used to perform a batch of correlation analysis of each miRNA, with corresponding target genes, and the three genes with the largest absolute negative correlation were retained as the most likely targets. Additionally, at least three potential target genes from miRTarBase, which is co-expressed with miRNAs, were considered as equally important and were subjected to Cytoscape (version 3.7.2) for identification of miRNA–target genes co-expression network analysis (Supplementary Figure S2).

### Functional Enrichment Analysis

The potential target genes that were negatively correlated with miRNAs in TCGA, as well as the coding sequences for mRNA and DNA methylation sites, were used for functional enrichment analysis using the Kyoto Encyclopedia of Genes and Genomes (KEGG) pathway and Gene Ontology (GO) using DAVID 6.8 (Supplementary Figure S2). Functional enrichment analysis indicates why the gene network produces images on the survival of GC from a molecular mechanism. Visualization was then done using the “ggplot2” package implemented in R.

### Statistical Analysis

The patients were divided into low-risk and high-risk groups by the median GS as the cutoff point. Survival estimates were obtained according to the Kaplan–Meier method and compared using the log-rank test. Variables that reached significance, with *P* < 0.05, were entered into the multivariable analyses using the Cox proportional hazards model, with an entry stepwise approach to identify covariates associated with increased all-cause mortality, and then hazard ratio with 95% confidence intervals (CIs) of each variable was achieved. All the statistical significance values were set as two-sided (*P* < 0.05). LASSO Cox regression was performed through the “glmnet” package. Time-dependent ROC analysis at different follow-up times was implemented using the “timeROC” package of R project in order to further expound the performance of different GS models, and DCA was used to compare their clinical use by “rmda” package. Finally, nomogram based on the Cox regression model was constructed using the “rms” package. C-index and calibration to calculate the discrimination and the stability of these models were performed using c-statistics and Bootstrap sample. Harrell’s concordance index (C-index) indicated a better prognostic model if its value was closer to 1, and the calibration diagram showed that the better the prediction if the closer the correction line was to the diagonal. All statistical methods are applied to both the training group and the validation group. Statistical analyses were performed using SPSS statistical software (version 18.0) and R software (version 3.5.3).

## Results

### Patient Characteristics

**FIGURE 2:**
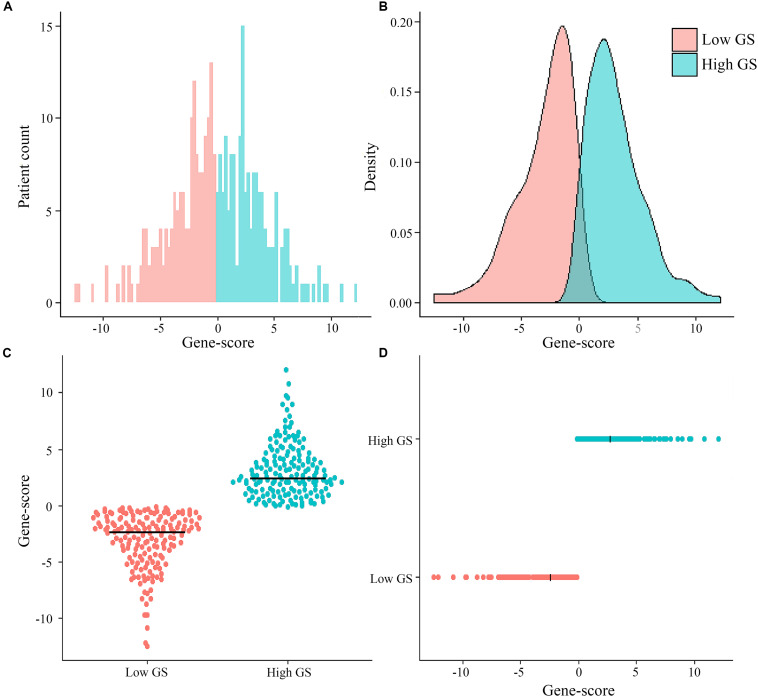
Distribution of patient cases and density based on genomics score (GS) in the total The Cancer Genome Atlas cohort **(A,B)**. Scatter plots of genomics scores regarding the classification of low and high GS **(C,D)**, and the bold line represents the median.

Among the 329 GC patients analyzed in this study, 212 (64.4%) were male, whereas 117 (35.6%) were female. The average age of the entire study population was 65.0 ± 10.6 years. In terms of pathological stage, 38 (11.6%) cases were identified as stage I, 117 (35.6%) were stage II, 155 (47.1%) were stage III, and 19 (5.8%) were at stage IV. With regards to treatment, 303 (92.1%) patients received surgery (280 cases of R0 surgery, 14 R1, and nine R2), whereas 146 (44.4%) were subjected to fluorouracil-based chemotherapy. The genomic nomogram classified 165 samples into low GS (GS ≤ -0.137) and 164 into high GS (GS > -0.137) groups based on the median cutoff (Figure 2). A detailed description of tumor location, pathology grade, and Lauren classification is outlined in Supplementary Table S1, while a heat map of the genomic scores layered by clinicopathological

factors is illustrated in Supplementary Figure S4. The median (mean; 95% CI) survival time for OS was 1,043 (670.2–1,415.8) days in the total cohort, 466 (370.6–561.4) days in the high-GS group, and 2,613 (mean; 2209.4–2017.5) days in the low-GS group (Supplementary Figure S5). Toward the last follow-up, a total of 129 deaths and 200 censoring were recorded. The estimated cumulative 1-, 3-, and 5-year OS in the total cohort were 78.9, 48.4, and 36.7%, respectively, although these rates increase to 95.1, 74.2, and 68.5%, respectively, in the low-GS group. Conversely, the 1-, 3-, and 5-year OS decreased to 63.3, 23.6, and 15.4%, respectively, in the high-GS group. The baseline information of the validation cohort is also listed in Supplementary Table S1 and Supplementary Figure S6.

### Survival Analysis

**FIGURE 3:**
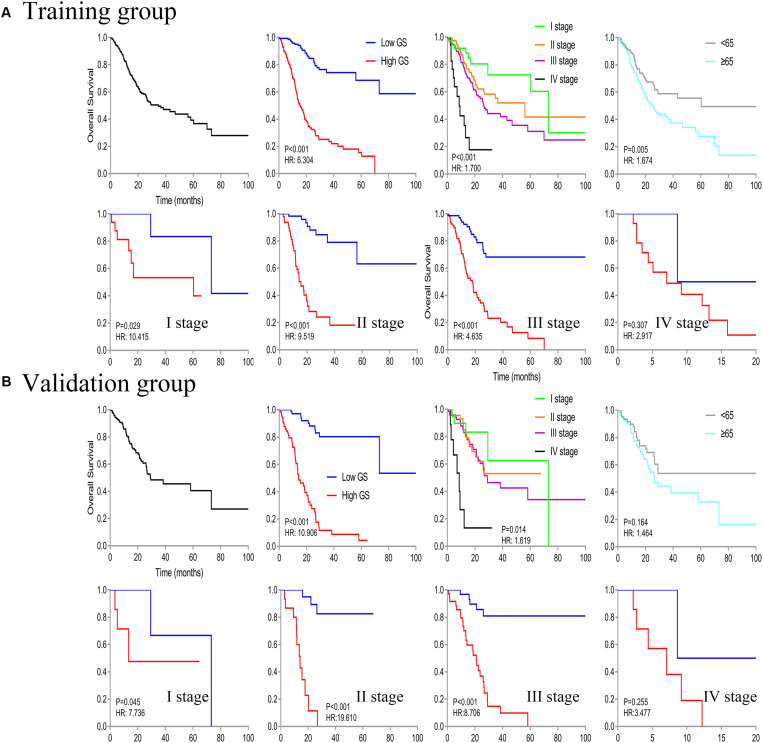
Kaplan–Meier curve of overall survival in all patients, then stratified by genomics score (GS), pathological stage, and age. Survival analysis in the low- and high-GS groups was further divided based on stages (stages I–IV).

We identified a basic genome-wide network comprising seven miRNAs, eight mRNAs, and 19 DNA methylation sites as the prognostic factor for OS, from hundreds of thousands of univariate Cox regression and LASSO analyses. This network was then classified as other models in the training and the validation groups. Among the 34 features identified, poor prognosis was significantly associated with a high expression of seven miRNAs (hsa-mir-100, hsa-mir-1304, hsa-mir-136, hsa-mir-193b, hsa-mir-22, hsa-mir-653, and hsa-mir-6808), six mRNAs (NRP1|8829, RNF144A|9781, ZNF22|7570, DUSP1|1843, CPNE8|144402, MAGED1|9500, and LOC91450|91450), and seven DNA methylation sites (cg07020967, cg08859156, cg12485556, cg15861578, cg15861578, cg25161386, and cg22740006). Conversely, poor prognosis was strongly associated with a low expression of SOX14|8403 and 12 DNA methylation sites, including cg02223323, cg00481239, cg14791193, cg15486740, cg20100408, cg20350671, cg22395807, cg24361571, cg25361506, cg22813794, cg26014401, and cg26856948 (Table 2). Univariate analysis performed on clinical characteristics revealed a significant association between age, pathological stage, TNM, and surgery with OS (Table 1). On the other hand, results from multivariable Cox regression showed that age, pathological stage, and GS were significantly associated with all-cause mortality in GC (Table 1 and Figure 3). Furthermore, stratification of the pathological stage (I, II, III, and IV) revealed significant differences in survival rates between the high-GS and the low-GS groups (Figure 3). A similar result was found when the data were stratified by demographic variables (sex and age), clinical characteristics (primary site, grade, and Lauren classification) as well as treatments (surgery and chemotherapy; Supplementary Figures S7, S8). On the other hand, categorizing GS into high or low groups, using the median value across different models, indicated that the genomics nomogram had the highest HR value. Interestingly, HR was almost equal to miRNAs + methylation, mRNA + methylation, Cox-model 1, and Cox-model 2 nomograms, which contained fewer gene features. Moreover, the HR value showed a marked decrease in miRNAs, mRNA, methylation, and miRNAs + mRNA nomograms, which included the least characteristics (Table 3).

**TABLE 1:** Univariable and multivariable analyses of the genomics score and the clinicopathological characteristics for overall survival in the training group and the validation group.

**Variables**	**Training group, *n* = 329**						**Validation group, *n* = 150**					
	**Univariable analysis**			**Multivariable analysis**			**Univariable analysis**			**Multivariable analysis**		
	**HR**	**95% CI**	***P* value**	**HR**	**95% CI**	***P* value**	**HR**	**95% CI**	***P* value**	**HR**	**95% CI**	***P* value**
**Age at diagnosis, tears**												
<65	1	1	NA	1	1	NA	1	1	NA	1	1	NA
≥65	1.674	1.167–2.402	0.005	2.043	1.407–2.966	<0.001	1.464	0.856–2.505	0.164	2.029	1.086–3.789	0.026
**Pathological stage**												
I	1	1	NA	1	1	NA	1	1	NA	1	1	NA
II	1.627	0.784–3.377	0.192	1.644	0.777–3.481	0.194	1.412	0.510–3.905	0.507	1.377	0.480–3.951	0.552
III	2.240	1.116–4.496	0.023	1.880	0.924–3.825	0.082	1.637	0.635–4.219	0.308	1.926	1.344–2.487	0.878
IV	7.801	3.247–18.745	<0.001	5.119	1.832–14.303	0.002	8.106	2.527–26.005	<0.001	9.364	2.267–38.672	0.002
**Surgery**												
R0	1	1	NA	1	1	NA	1	1	NA	1	1	NA
R1	1.556	0.755–3.209	0.231	1.214	0.578–2.547	0.608	1.240	0.286–5.372	0.774	0.937	0.209–4.209	0.932
R2	6.944	3.163–15.246	<0.001	1.686	0.615–4.621	0.310	12.906	3.796–43.886	<0.001	1.316	0.285–6.075	0.725
Unknown	2.373	1.347–4.182	0.003	2.006	1.115–3.607	0.020	2.309	1.030–5.175	0.042	2.000	0.852–4.693	0.111
**Genomics score^a^**												
Low	1	1	NA	1	1	NA	1	1	NA	1	1	NA
High	6.304	4.079–9.744	<0.001	6.093	3.910–9.493	<0.001	10.906	5.452–21.817	<0.001	12.886	6.158–26.963	0.000
**T staging**												
T1	1	1	NA				1	1	NA			
T2	7.604	1.022–56.585	0.048				5.008	0.638–39.304	0.125			
T3	7.278	1.003–52.802	0.050				3.895	0.524–28.976	0.184			
T4	9.473	1.312–68.368	0.026				3.951	0.536–29.143	0.178			
**N staging**												
N0	1	1	NA				1	1	NA			
N1	1.424	0.869–2.335	0.161				1.563	0.722–3.393	0.257			
N2	1.642	0.930–2.898	0.087				1.612	0.658–3.953	0.296			
N3	2.200	1.369–3.535	0.001				2.509	1.252–5.029	0.009			
**M staging**												
M0	1	1	NA				1	1	NA			
M1	4.224	2.309–7.726	<0.001				5.499	2.446–12.364	<0.001			
**Sex**												
Female	1	1	NA				1	1	NA			
Male	1.449	0.989–2.123	0.057				1.126	0.648–1.956	0.674			
**Primary site**												
Cardia	1	1	NA				1	1	NA			
Fundus/body	0.844	0.543–1.312	0.451				0.605	0.320–1.144	0.122			
Antrum	0.822	0.530–1.274	0.380				0.763	0.394–1.476	0.422			
Unknown	0.183	0.025–1.343	0.095				0.152	0.003–0.254	0.976			
**Pathology grade**												
I–II	1	1	NA				1	1	NA			
III–IV	1.361	0.939–1.971	0.103				1.590	0.902–2.805	0.109			
Unknown	1.881	0.673–5.257	0.228				2.534	0.854–7.524	0.094			
**Lauren classification**												
Intestinal type	1	1	NA				1	1	NA			
Diffused type	1.245	0.805–1.925	0.326				1.416	0.728–2.756	0.305			
Unknown	1.156	0.770–1.736	0.484				1.923	1.061–3.485	0.031			
**Chemotherapy**												
Yes	1	1	NA				1	1	NA			
No	1.305	0.919–1.852	0.136				1.361	0.806–2.299	0.249			

*^*a*^Based on 34 biomarkers: seven miRNAs, eight mRNAs, and 19 DNA methylation sites.*

**TABLE 2:** miRNAs, mRNA, and DNA methylation whose expression levels showed a significant association with overall survival in least absolute shrinkage and selection operator.

**Molecular (probe)**	**ID (reference gene)**	**Coefficient**	**HR**	**95% CI**	**SE**	***z* value**	***p* value**
miRNAs	hsa-mir-100	0.234	1.263	1.102–1.449	0.070	3.345	<0.001
	hsa-mir-1304	0.113	1.120	1.006–1.247	0.055	2.060	0.039
	hsa-mir-136	0.235	1.265	1.097–1.458	0.072	3.243	0.001
	hsa-mir-193b	0.241	1.272	1.116–1.450	0.067	3.612	<0.001
	hsa-mir-22	0.248	1.281	1.101–1.490	0.077	3.210	0.001
	hsa-mir-653	0.148	1.160	1.046–1.287	0.053	2.804	0.005
	hsa-mir-6808	0.180	1.197	1.027–1.396	0.078	2.297	0.022
mRNA	NRP1|8829	0.291	1.338	1.178–1.519	0.065	4.492	<0.001
	RNF144A|9781	0.313	1.367	1.186–1.576	0.073	4.313	<0.001
	ZNF22|7570	0.302	1.353	1.160–1.579	0.079	3.843	<0.001
	SOX14|8403	–0.464	0.629	0.470–0.841	0.148	–3.126	0.002
	DUSP1|1843	0.360	1.434	1.240–1.657	0.074	4.874	<0.001
	CPNE8|144402	0.342	1.407	1.203–1.646	0.080	4.269	<0.001
	MAGED1|9500	0.291	1.338	1.165–1.537	0.071	4.112	<0.001
	LOC91450|91450	0.278	1.320	1.156–1.509	0.068	4.083	<0.001
cg02223323	MAP7D2	–0.360	0.697	0.590–0.824	0.085	–4.225	<0.001
cg00481239	SHC4;EID1	–0.668	0.513	0.338–0.777	0.212	–3.153	0.002
cg07020967	TMEM117	0.380	1.462	1.215–1.759	0.094	4.026	<0.001
cg08859156	RPS4X	0.409	1.505	1.305–1.736	0.073	5.609	<0.001
cg12485556	PREP	0.435	1.545	1.196–1.994	0.130	3.333	<0.001
cg14791193	C1orf144	–0.400	0.671	0.581–0.773	0.073	–5.496	<0.001
cg15861578	ZC3H10	0.329	1.390	1.169–1.652	0.088	3.731	<0.001
cg15486740	ACOT13;TTRAP	–0.363	0.695	0.573–0.843	0.098	–3.697	<0.001
cg20100408	HLA-DPB1	–0.357	0.700	0.605–0.810	0.074	–4.799	<0.001
cg20350671	IL1RAPL1	–0.390	0.677	0.561–0.817	0.096	–4.080	<0.001
cg22395807	ATXN10	–0.443	0.643	0.476–0.867	0.153	–2.898	0.004
cg24361571	MIR365-2	–0.340	0.712	0.611–0.829	0.078	–4.374	<0.001
cg25361506	Unconfirmed	–0.362	0.696	0.587–0.825	0.087	–4.167	<0.001
cg25622155	Unconfirmed	0.331	1.392	1.176–1.647	0.086	3.851	<0.001
cg25161386	NUFIP2	0.305	1.357	1.159–1.590	0.081	3.787	<0.001
cg22740006	PC;LRFN4	0.342	1.407	1.149–1.723	0.103	3.303	<0.001
cg22813794	STYXL1;MDH2	–0.351	0.704	0.508–0.976	0.166	–2.106	0.035
cg26014401	Unconfirmed	–0.430	0.651	0.539–0.786	0.097	–4.453	<0.001
cg26856948	GOLGA3	–0.334	0.716	0.609–0.842	0.083	–4.042	<0.001

**TABLE 3:** Comparison of different genomics score models (based on the median value) for overall survival in the training group and the validation group.

**Variables**	**Training group, *n* = 329**			**Validation group, *n* = 150**		
	**Hazard ratio**	**95% CI**	***P* value**	**Hazard ratio**	**95% CI**	***P* value**
**Genomics nomogram**						
Age	1.897	1.322–2.724	0.001	1.547	0.903–2.652	0.112
Pathological stage	1.489	1.155–1.920	0.002	1.267	0.846–1.897	0.252
Genomics score^a^	6.153	3.971–9.535	<0.001	10.141	5.011–20.520	<0.001
**Clinical nomogram**						
Age	1.760	1.225–2.528	<0.002	1.778	1.025–3.085	0.041
Pathological stage	1.754	1.349–2.282	<0.001	1.788	1.194–2.677	0.005
**miRNAs nomogram**						
Age	1.664	1.157–2.392	0.006	1.597	0.917–2.780	0.098
Pathological stage	1.748	1.345–2.273	<0.001	1.771	1.181–2.656	0.006
Genomics score^b^	2.011	1.402–2.883	<0.001	2.474	1.416–4.324	0.001
**mRNA nomogram**						
Age	2.140	1.484–3.086	<0.001	1.862	1.076–3.222	0.026
Pathological stage	1.716	1.314–2.241	<0.001	1.610	1.058–2.449	0.026
Genomics score^c^	3.222	2.209–4.700	<0.001	4.834	2.671–8.781	<0.001
**Methylation nomogram**						
Age	1.798	1.253–2.580	0.001	1.834	1.070–3.144	0.027
Pathological stage	1.599	1.231–2.078	<0.001	1.362	0.903–2.055	0.140
Genomics score^d^	4.627	3.058–7.002	<0.001	7.271	3.694–14.313	<0.001
**miRNAs + methylation nomogram**						
Age	1.750	1.220–2.511	0.002	1.692	0.987–2.899	0.056
Pathological stage	1.539	1.193–1.986	0.001	1.414	0.954–2.096	0.084
Genomics score^e^	5.009	3.291–7.624	<0.001	9.080	4.399–18.739	<0.001
**miRNAs + mRNA nomogram**						
Age	1.932	1.343–2.778	<0.001	1.824	1.057–3.148	0.031
Pathological stage	1.676	1.291–2.177	<0.001	1.546	1.027–2.326	0.037
Genomics score^f^	2.894	1.993–4.203	<0.001	3.431	1.969–5.979	<0.001
**mRNA + methylation nomogram**						
Age	1.939	1.351–2.784	<0.001	1.768	1.032–0.3.031	0.038
Pathological stage	1.523	1.181–1.965	0.001	1.322	0.882–1.979	0.176
Genomics score^g^	5.050	3.330–7.658	<0.001	7.553	3.911–14.586	<0.001
**Cox-model 1 nomogram**						
Age	1.908	1.329–2.740	<0.001	1.878	1.091–3.233	0.023
Pathological stage	1.642	1.276–2.112	<0.001	1.688	1.126–2.530	0.011
Genomics score^h^	5.034	3.334–7.601	<0.001	9.334	4.671–18.652	<0.001
**Cox-model 2 nomogram**						
Age	2.033	1.415–2.921	<0.001	1.777	1.030–3.067	0.039
Pathological stage	1.647	1.261–2.151	<0.001	1.722	1.142–2.595	0.009
Genomics score^i^	5.481	3.602–8.341	<0.001	8.679	4.347–17.325	<0.001

*^*a*^Based on 34 biomarkers (seven miRNAs, eight mRNA, and 19 DNA methylation sites). ^*b*^Based on seven miRNAs. ^*c*^Based on eight mRNA. ^*d*^Based on 19 DNA methylation sites. ^*e*^Based on seven miRNAs + 19 DNA methylation sites. ^*f*^Based on seven miRNAs + eight mRNA. ^*g*^Based on eight mRNA + 19 DNA methylation sites. ^*h*^Based on two miRNAs + six mRNA + nine DNA methylation sites. ^*i*^Based on one miRNAs + one mRNA + seven DNA methylation sites.*

### Nomograms Based on Genome-Wide Network

**FIGURE 4:**
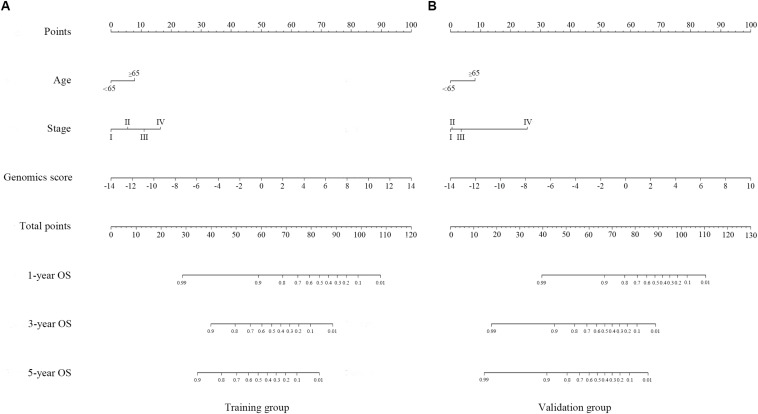
Genomics nomogram to predict the probability of 1-, 3-, and 5-year overall survival (OS) in the training cohort **(A)** and the validation cohort **(B)**: to determine how many points for each variable to the probability of OS, locate the variable on its axis, draw a line straight upward to the point axis, repeat this process for each variable, sum up the points achieved for each of the risk factors, locate the final sum on the total point axis, and draw a line straight down to find the patient’s probability of OS.

**FIGURE 5:**
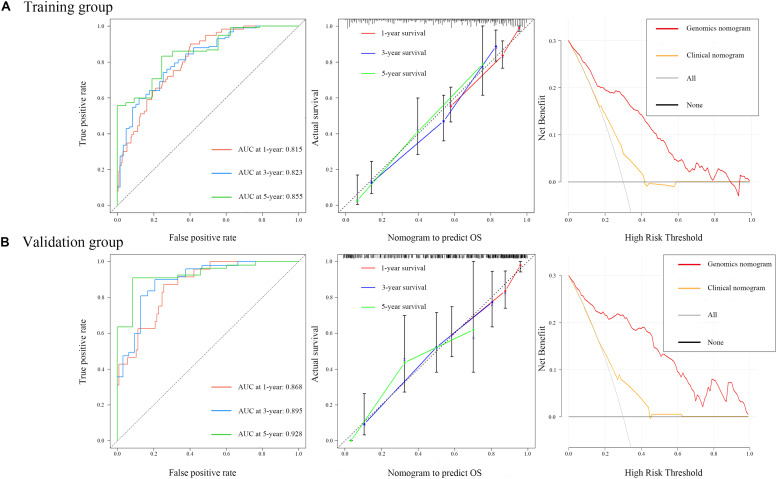
Time-dependent receiver operating characteristic curves on 1, 3, and 5 years of genomics nomogram. Calibration plot showing 1-, 3-, and 5-year overall survival (OS) probability: the nomogram-estimated OS is plotted on the x-axis, and the actual OS is plotted on the *y*-axis. The diagonal dotted line is a perfect estimation by an ideal model, in which the estimated outcome perfectly corresponds to the actual outcome. The solid line is the performance of the nomogram: A closer alignment with the diagonal dotted line represents a better estimation. Decision curve analysis for genomics nomogram and clinical nomogram: the y-axis measures the net benefit. The gray line or the horizontal black line represents a follow-up of all patients or no patients. The model makes more benefit with a higher position in curve.

A genomics nomogram was first constructed based on the genome-wide network, comprising 34 gene features (Figure 4). To obtain a more concise and effective nomogram, we also built a Cox-model 1 (17 gene features) and Cox-model 2 (nine gene features) nomograms (Supplementary Figures S9, S10). Next, a clinical nomogram, based on stage and age, was built as a control (Supplementary Figure S11). Thereafter, we performed internal and external validation to evaluate the feasibility of all nomograms using a three-grouped random bootstrap sampling (Figure 5 and Supplementary Figures S9–S11). We observed good predictive performance in the first three nomograms, but not in the simple clinical model.

### Validation of the Nomograms Using ROC and DCA

**FIGURE 6:**
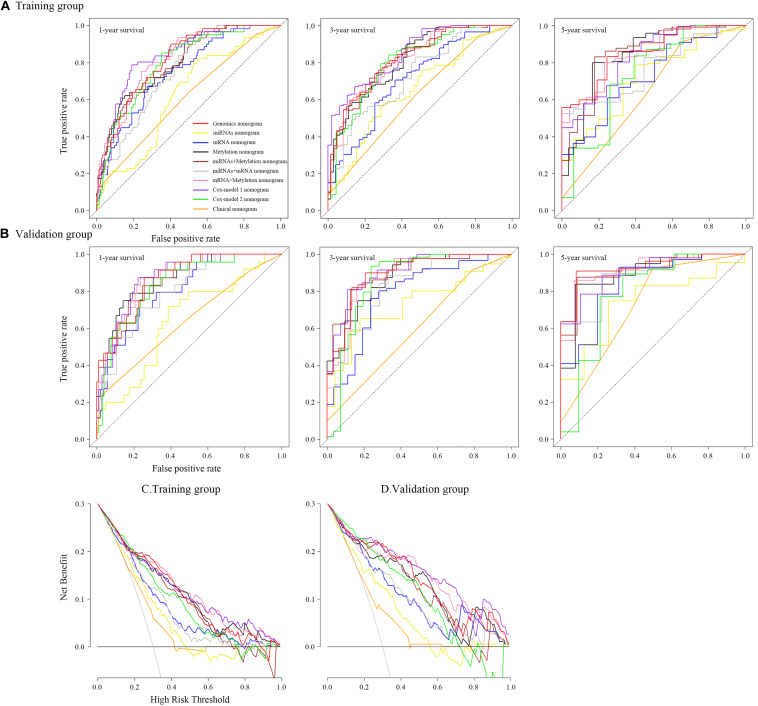
Time-dependent receiver operating characteristic curves on 1, 3, and 5 years of each nomogram and decision curve analysis for each nomogram.

To ensure a good comparison across different GS nomograms, we performed a time-dependent ROC (at 1, 3, and 5 years of follow-up) as well as DCA. In the validation group, genomics nomogram revealed the best comprehensive performance, with 1-, 3-, and 5-year area under the curve (AUC) values of 0.868, 0.895, and 0.928, respectively (Table 4), and Cox-model 1, miRNAs + methylation, and mRNA + methylation nomograms had a comparable performance, with 1-, 3-, and 5-year AUC values of 0.856–0.873, 0.884–0.905, and 0.907–0.919, respectively, but it had fewer biomarkers (Table 4). Although the Cox-model 2 nomograms had the least biomarkers, including miRNA, mRNA, and DNA methylation sites, it had a relatively poor performance with 1-, 3-, and 5-year AUC values of 0.835, 0.859, and 0.785, respectively. Besides that, the miRNA, mRNA, methylation, miRNAs + methylation, and miRNAs + mRNA nomograms recorded 1-, 3-, and 5-year AUC values of 0.729–0.877, 0.656–0.805, and 0.721–0.894, respectively. Finally, we found that, compared to miRNA (0.641, 0.729, and 0.736) and mRNA nomogram (0.806, 0.785, and 0.843), methylation nomogram had higher 1-, 3-, and 5-year AUC values of 0.866, 0.877, and 0.894. Nevertheless, all of them showed better performance than the clinical nomogram, which recorded 1-, 3-, and 5-year AUC values of 0.638, 0.598, and 0.721, respectively (Figures 6A,B and Supplementary Figure S12). The C-index based on different nomograms exhibited a similar effect (Supplementary Table S8). Additionally, DCA showed that the genomics, Cox-model 1, mRNA + methylation, and methylation nomograms had a significant net benefit compared to other GS models and the clinical nomogram (Figures 6C,D).

**TABLE 4:** The area under the curve (AUC) values of different genomics score models in the training group and the validation group.

**Models**	**Training group, *n* = 329**			**Validation group, *n* = 150**		
	**1-year OS**	**3-year OS**	**5-year OS**	**1-year OS**	**3-year OS**	**5-year OS**
	**AUC (95% CI)**	**AUC (95% CI)**	**AUC (95% CI)**	**AUC (95% CI)**	**AUC (95% CI)**	**AUC (95% CI)**
Genomics nomogram	0.815 (0.787–0.843)	0.823 (0.785–0.861)	0.855 (0.799–0.911)	0.868 (0.832–0.900)	0.895 (0.851–0.939)	0.928 (0.886–0.970)
Clinical nomogram	0.609 (0.571–0.647)	0.615 (0.573–0.657)	0.642 (0.582–0.702)	0.638 (0.577–0.699)	0.598 (0.528–0.659)	0.721 (0.626–0.816)
miRNAs nomogram	0.621 (0.582–0.660)	0.656 (0.610–0.703)	0.717 (0.650–0.779)	0.641 (0.581–0.701)	0.729 (0.670–0.788	0.736 (0.656–0.817)
mRNA nomogram	0.747 (0.713–0.781)	0.711 (0.666–0.756)	0.728 (0.65.6–0.79.8)	0.806 (0.761–0.851)	0.785 (0.724–0.846)	0.843 (0.766–0.918)
Methylation nomogram	0.799 (0.768–0.830)	0.813 (0.774–0.852)	0.845 (0.781–0.909)	0.866 (0.827–0.905)	0.877 (0.830–0.923)	0.894 (0.821–0.966)
miRNAs + methylation nomogram	0.796 (0.765–0.827)	0.819 (0.781–0.857)	0.850 (0.787–0.911)	0.856 (0.817–0.895)	0.895 (0.854–0.939)	0.908 (0.856–0.961)
miRNAs + mRNA nomogram	0.743 (0.710–0.776)	0.731 (0.687–0.775)	0.771 (0.707–0.835)	0.803 (0.758–0.848)	0.825 (0.772–0.878)	0.883 (0.826–0.939)
mRNA + methylation nomogram	0.819 (0.791–0.847)	0.818 (0.780–0.856)	0.849 (0.794–0.904)	0.873 (0.837–0.909)	0.884 (0.836–0.932)	0.919 (0.867–0.971)
Cox-model 1 nomogram	0.833 (0.804–0.862)	0.851 (0.821–0.881)	0.833 (0.778–0.888)	0.869 (0.832–0.906)	0.905 (0.866–0.944)	0.907 (0.858–0.956)
Cox-model 2 nomogram	0.795 (0.764–0.826)	0.805 (0.767–0.843)	0.736 (0.662–0.810)	0.835 (0.793–0.877)	0.859 (0.797–0.921)	0.785 (0.667–0.903)

### Potential miRNA Target Genes

A total of 72 hsa-mir-22, 39 hsa-mir-100, 56 hsa-mir-136, 58 hsa-mir-193b, 23 hsa-mir-653, 96 hsa-mir-1304, and 285 hsa-mir-6808 potential target genes were identified from the miRTarBase, miRDB, and TargetScan databases (Supplementary Figure S14). We then performed a correlation analysis between each target gene and miRNAs and finally generated a miRNA–potential target gene plot (Supplementary Figure S15A) as well as a miRNA–target gene co-expression network (Supplementary Figure S15B) using Cytoscape.

### Functional Analysis of Genome-Wide Network

**FIGURE 7:**
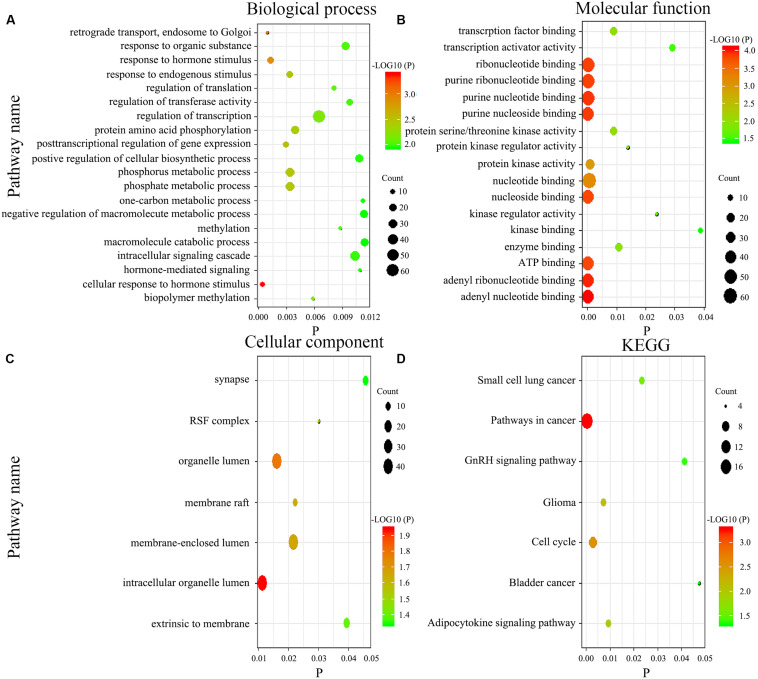
Gene enrichment analysis and Kyoto Encyclopedia of Genes and Genomes (KEGG) pathways for the genome-wide network. The biological process **(A)**, molecular function **(B)**, cellular component **(C)**, and KEGG pathways **(D)**.

We imported the 301 potential target genes, mRNA, and DNA methylation site-coding sequences, identified above, into DAVID for KEGG and GO analyses and identified biological processes, molecular functions as well as cellular components (Figures 7A–C). Their corresponding KEGG pathways were also plotted (Figure 7D).

## Discussion

GC can be divided into two types or four main categories, according to the Lauren and World Health Organization (WHO) classifications (Lauren, 1965; Nagtegaal et al., 2019), although neither of these classifications is based on molecular markers. In the last decade, however, three novel molecular-based classification systems have been suggested for GC. The Singapore-Duke Group was the first to describe a classification with two intrinsic genomic subtypes, G-INT, and G-DIF, which had different gene expression (Tan et al., 2011; Serra et al., 2019). The subtypes have different levels of resistance to various chemotherapy drugs and show limited prognostic value. Later, TCGA used molecular evaluation to propose a new classification with four subtypes: EBV, MSI, GS, and CIN. The identification of these subtypes has provided a roadmap for patient stratification as well as targeted therapeutic trials (Cancer Genome Atlas Research, 2014). However, initial data on disease outcomes from this cohort did not show differences in survival among the four groups. A series of positive studies on prognosis based on TCGA classification was also reported (Sohn et al., 2017). In addition, the Asian Cancer Research group divided GC into four subtypes, MSI, EMT, MSS/TP53+, and MSS/TP53-, based on gene expression data and found significantly different survival outcomes across them (Cristescu et al., 2015; Serra et al., 2019). Despite the significant milestones of these studies, they are all mainly based on the analysis of gene expression (mRNA). Besides that, a 2019 study proposed a five-miRNA model, while it had a C-index of 0.72 only (Zhang et al., 2019). In the current study, we included methylation data and performed functional enrichment analysis, making our work stronger. The aforementioned classifications are also complicated and need further optimization to increase clinical applicability. Furthermore, they focused on typing and finding new targets, whereas our study reports on prognostic analysis.

Some of the biomarkers we identified herein, including hsa-mir-22, hsa-mir-100, hsa-mir-136, hsa-mir-193b, hsa-mir-1304, NRP1, DUSP1, and MAP7D2 (cg02223323), have previously been reported in GC (Grandclement and Borg, 2011; Chen et al., 2014; Mu et al., 2014; Zuo et al., 2015; Zheng et al., 2017; Chen et al., 2018; Kurata and Lin, 2018; Liu K.T. et al., 2018; Song et al., 2018; Teng et al., 2018; Liu et al., 2019; Pan et al., 2019; Wang et al., 2019). Others, such as CPNE8, MAGED1, RNF144A, SOX14, ACOT13 (cg15486740), EID1 (cg00481239), RPS4X (cg08859156), and TTRAP (cg15486740), have been identified in various tumors other than GC (Kamio et al., 2010; Zeng et al., 2012; Zhou et al., 2013; Kuang et al., 2017; Stanisavljevic et al., 2017; Liu X. et al., 2018; Tosic et al., 2018; Nagasawa et al., 2019; Yang et al., 2019). The remaining biomarkers, including hsa-mir-653, hsa-mir-6808, LOC91450, ZNF22, C1orf144 (cg14791193), GOLGA3 (cg26856948), HLA-DPB1 (cg20100408), LRFN4 (cg22740006), MDH2 (cg22813794), MIR365-2 (cg24361571), NUFIP2 (cg25161386), PREP (cg12485556), STYXL1 (cg22813794), TMEM117 (cg07020967), ZC3H10 (ZC3H10), IL1RAPL1 (cg20350671), PC (cg22740006), SHC4 (cg00481239), and ATXN10 (cg22395807), have not been previously reported.

Currently, focus has been directed on identifying prognostic miRNAs for GC. Particularly, one miRNA can regulate multiple targets, while multiple miRNAs can regulate a single mRNA. Therefore, a single miRNA may play an opposite role in cancer progression by regulating different target genes. For example, Mir-22 and Mir-100 were found to be tumor suppressors in various cancers, including GC (Chen et al., 2014; Zuo et al., 2015). Similarly, a high expression of Mir-136 was found to promote proliferation and invasion in GC cell lines by inhibiting PTEN expression (Chen et al., 2018), while a contrasting result was reported when HOXC10 was targeted (Zheng et al., 2017). Similarly, Mir-193b reportedly induced GC proliferation or apoptosis by mediating different mRNA expressions (Mu et al., 2014; Song et al., 2018), whereas a high Mir-1304 expression in GC was reported as a negative predictor for prognosis of lung and thyroid cancers (Kurata and Lin, 2018; Liu et al., 2019; Pan et al., 2019). However, the function of Mir-653 and Mir-6808 has not been previously reported. In the current study, we found an association between a high expression of all miRNAs and poor survival. Different outcomes may be observed in our study, relative to previous reports, owing to the huge number of corresponding miRNA target genes herein and lack of evidence on their role in GC development.

Messenger RNAs have been reported to play an essential role in GC cancer. For example, high NRP1 expression and hypermethylation were associated with poor GC prognosis (Wang et al., 2019), whereas another study indicated that it could be an anti-tumor target (Grandclement and Borg, 2011). In addition, high DUSP1 expression levels were found to promote progression, drug resistance, and poor prognosis of GC (Teng et al., 2018). On the other hand, SOX14 showed opposite prognostic values in cervical cancer and leukemia, with anti-tumor and carcinogenic effects, respectively (Stanisavljevic et al., 2017; Tosic et al., 2018). Studies have also implicated CPNE8, MAGED1, and RNF144A in ovarian and breast cancers (Zeng et al., 2012; Nagasawa et al., 2019; Yang et al., 2019). However, LOC91450 and ZNF22 have not been reported in cancer.

Accumulating evidence indicates that DNA methylation plays a significant role in cancer progression. However, only a handful of studies have described the relationship between levels of single-site methylation and GC prognosis. Particularly, high expressions of MAP7D2, ACOT13, EID1, RPS4X, and TTRAP have been associated with poor prognosis in gastric, lung, and pancreatic cancers as well as hepatic carcinoma, respectively, while a high TTRAP expression reportedly inhibits the growth of osteosarcoma (Kamio et al., 2010; Zhou et al., 2013; Kuang et al., 2017; Liu K.T. et al., 2018; Liu X. et al., 2018). Notably, the relationship between methylation levels and corresponding gene expression profiles is unknown, necessitating further research. Furthermore, the remaining DNA methylation sites and their corresponding genes have not been reported. Lastly, no study has described the prognostic significance using a genome-wide network.

Last but not least, in general, no study concerning their prognostic significance as a genome-wide network has been reported yet.

Tumorigenesis involves multiple interacting biological processes. In addition, an integrated genetic network is better at reflecting intra-tumor heterogeneity compared to a single biomarker. In the current study, we identified a novel, prognostic, signature genome-wide network, consisting of seven miRNAs, eight mRNA, and 19 DNA methylation sites after screening the entire TCGA cohort using training and random cohorts. This network was further divided into several other models.

Our results revealed that the integrative signature was an independent prognostic factor for survival in GC patients and performed better than any single biomarker or clinical characteristic. Moreover, stratification by other clinicopathological features, such as stage, age, sex, primary site, pathology grade, Lauren classification, and treatments, revealed significantly different prognosis values based on different GSs. In addition, staging was still an effective prognostic factor after dividing into low- and high-genomics-score groups, suggesting that GS and traditional staging can complement each other, and the genetic network could add prognostic value to traditional staging. Exclusion of patients with I staging showed that chemotherapy is a significant prognostic factor because I staging does not always need additional chemotherapy for effective prognosis.

We also developed and validated nomograms based on the GS. Particularly, results from ROC and DCA indicated that all of them had significantly better predictive performances than the traditional clinical nomogram. Comprehensive property (similar C-index) was not significantly different in genomics nomogram and Cox-model 1 nomogram, and compared to the genomics nomogram, Cox-model 1 nomogram had fewer biomarkers. In addition, Cox-model 1 nomogram performed well, with a higher positive net reclassification improvement (NRI). Therefore, Cox-model 1 nomogram might be more suitable for clinical application, which deserved further study. Besides that, the Cox-model 2 nomogram had the least feature (nine biomarkers) including miRNAs, mRNA, and DNA methylation sites for constructing a genome-wide network, while it had a lower C-index and a negative NRI. The other six models showed a relatively poor performance in ROC or DCA, with limited application value. What is more, it is possible that DNA methylation was the highest contributor to the survival prediction of this gene network. We suspect that this may be related to the larger number of DNA methylation sites compared to miRNA and mRNA.

We adopted GO and KEGG analyses to assess the influence of genome-wide network in the prognosis of GC. Generally, biological processes mainly involve various biological functions, such as methylation, phosphorylation, and endocrine regulation. Methylation pathway was related to the occurrence and the development of GC, which was consistent with our results. Besides that, functional enrichment analysis revealed that phosphorylation pathway was significantly enriched as well, which got more and more attention these years. On the other hand, the main components of participation included organelles, cytomembranes, extrinsic to membranes, nuclear and synapses, whereas molecular functions comprise nucleoside, ATP, RNA, and transcription factor binding as well as activity of various enzymes. Abnormal cell composition is closely related to the development of tumor. The abnormal protein may act on the nucleus, membrane, or cell matrix, thereby leading to the progression of cancer, such as NRP1 protein (Wang et al., 2019). In the current study, KEGG analysis indicated that the gene network function was a relevant pathway in cancer, cell cycle, and adipocytokine signaling, while the other pathways had been reported in small cell lung and bladder cancers. Further experiments to reveal the biological function of this gene network are needed.

We also employed a series of complex statistical analyses to construct and validate a genome-wide network based on different biomarkers and then divided it into different models. We recommend the resulting GS despite it not being an absolute representative of tumor heterogeneity. This network could complement the deficiency of traditional staging and generate a more accurate prediction of survival rates in GC patients. Additionally, it effectively distinguishes patients who could benefit from chemotherapy, thereby reducing unnecessary treatments. It is also possible that the network could be used to identify novel therapeutic targets for GC, although this requires further investigation.

### Limitation

This study had several limitations. Firstly, information relating to patient co-morbidities and performance status was not available in the TCGA database. Secondly, the systemic chemotherapy regimens were not uniform, and most of them were based on fluoropyrimidines. Thirdly, the gene network contains too many biomarkers, increasing the difficulty of clinical use. Lastly, this was a retrospective study, without any independent external patient datasets as test. Despite some limitations, it was the first, to the best of our knowledge, to integrate miRNAs, mRNA, and DNA methylation sites as a genome-wide network to predict the OS of patients with GC, and we would try to design a validation in our hospital.

## Conclusion

In summary, we used a TCGA cohort to develop and validate a novel genome-wide network comprising seven miRNAs, eight mRNAs, and 19 DNA methylation sites for the prognosis of GC. A combination of GS and TNM staging enhances its prognostic value, proposing a more comprehensive sub-typing system. The developed network is expected to aid in predicting GC patients who may benefit from chemotherapy to some degree.

## Data Availability Statement

The datasets generated and analyzed during the current study are available in the TCGA database [https://portal.gdc.cancer.gov/].

## Ethics Statement

Since TCGA was a public-use database, no additional permission was required from the Ethics Committee. In addition, this study was deemed exempt from institutional review board approval by Nanfang Hospital of Southern Medical University (Guangzhou, China).

## Author Contributions

All the authors listed had made a substantial contribution to this work. YJ and GL put forward the conception and designed the study. WH, TL, and MZ collected and collated the data. ZS, HC, and ZH analyzed the data and wrote the manuscript together. HL, JY, and YH made contribution to proofread the article. Finally, all the authors took responsibility of the final manuscript and approved it for publication.

## Conflict of Interest

The authors declare that the research was conducted in the absence of any commercial or financial relationships that could be construed as a potential conflict of interest.

## Supplementary Material

FIGURE S1 Screening process for the genome-wide network.

FIGURE S2 Screening process for the Gene Ontology analysis and Kyoto Encyclopedia of Genes and Genomes pathways.

FIGURE S3 Least absolute shrinkage and selection operator Cox regression performed for miRNAs, mRNA, and DNA methylation sites.

FIGURE S4 A heat map based on genomic scores layered by clinicopathological factors.

FIGURE S5 Median survival for patients in the low-genomics score (GS) and the high-GS groups.

FIGURE S6 Distribution of patient cases and density based on genomics score in the validation group **(A,B)**. Scatter plots of genomics score (GS) regarding the classification of low- and high-GS **(C,D)**, and the bold line represents the median.

FIGURE S7 Kaplan–Meier curve of overall survival for the low- and the high-genomics-score groups divided by age (young and old), sex (male and female), primary site (cardia, fundus/body, and antrum), pathology grade (I–II and III–IV), Lauren classification (intestinal type and diffused type), and treatment (R0 surgery, R1 surgery, chemotherapy, and no chemotherapy) in the training group.

FIGURE S8 Kaplan–Meier curve of overall survival for the low- and the high-genomics-score groups divided by age (young and old), sex (male and female), primary site (cardia, fundus/body, and antrum), pathology grade (I-II and III-IV), Lauren classification (intestinal type and diffused type), and treatment (R0 surgery, R1 surgery, chemotherapy, and no chemotherapy) in the validation group.

FIGURE S9 Cox-model 1 nomogram and its calibration plot in the training group and the validation group.

FIGURE S10 Cox-model 2 nomogram and its calibration plot in the training group and the validation group.

FIGURE S11 Clinical nomogram and its calibration plot in the training group and the validation group.

FIGURE S12 Time-dependent receiver operating characteristic curves on 1, 3, and 5 years for each nomogram in the training group.

FIGURE S13 Time-dependent receiver operating characteristic curves on 1, 3, and 5 years for each nomogram in the validation group.

FIGURE S14 Venn diagrams for the target genes of each miRNA.

FIGURE S15 Three most potential target genes for each miRNA **(A)** and the miRNA–target genes co-expression network **(B)**.

FIGURE S16 Time-dependent receiver operating characteristic curves on 1, 3, and 5 years for each signature in the genome-wide network. Each color represents a different signature: red 2 (genomics nomogram), orange (clinical nomogram), yellow (hsa-mir-100), blue (hsa-mir-1304), black (hsa-mir-193b), brown (hsa-mir-22), gray (hsa-mir-136), pale violet red 1 (hsa-mir-653), purple 2 (hsa-mir-6808), green 2 (NRP1|8829), dark red (SOX14|8403), dark orchid 3 (CPNE8|144402), dark orange 2 (MAGED1|9500), cyan (RNF144A|9781), corn silk 4 (ZNF22|7570), hot pink 1 (DUSP1|1843), chartreuse (LOC91450|91450), aquamarine 2 (cg02223323), dark green (cg00481239), maroon 1 (cg07020967), pale green (cg08859156) gold (cg12485556), purple 1 (cg14791193), antique white (cg15861578), khaki 2 (cg15486740) sea green 2 (cg20100408), light sky blue (cg20350671), maroon (cg22395807), Indian red 2 (cg24361571), tan 1 (cg25361506), peach puff (cg25622155), turquoise 1 (cg25161386), orchid 2 (cg22740006), dark golden rod 2 (cg22813794), dark golden rod 4 (cg26014401), and violet red 1 (cg26856948).

FIGURE S17 Kaplan–Meier curve of overall survival in the low-genomics-score group stratified by clinical features (e.g., stage).

FIGURE S18 Kaplan-Meier curve of overall survival in the high-genomics-score group stratified by clinical features (e.g., stage).

FIGURE S19 Kaplan–Meier curve of overall survival in stages II and III patients.

TABLE S1 Descriptive statistics of gastric cancer patients.

TABLE S2 Multivariable analysis for miRNAs, mRNA, and DNA methylation sites and clinical characteristics.

TABLE S3 Multivariable analysis for miRNAs.

TABLE S4 Multivariable analysis for mRNA.

TABLE S5 Multivariable analysis for DNA methylation sites.

TABLE S6 The equation of different models.

TABLE S7 The C-index value of different models.

TABLE S8 The area under the curve values for each individual of the genome-wide network.
