# Supplementary material for: Genomics Score Based on Genome-Wide Network Analysis for Prediction of Survival in Gastric Cancer: A Novel Prognostic Signature
Source: Front Genet. 2020 Aug 6;11:835. doi: 10.3389/fgene.2020.00835 (PMC7423976; doi:10.3389/fgene.2020.00835)
Supplement: FIGURE S1 — Screening process for the genome-wide network. [file Data_Sheet_1.docx]

Supplementary Material

I. Supplementary tables: 8

II. Supplementary figures: 19

I.

| **Table S1. Descriptive statistics of GC patients** | | | | | | | |
| --- | --- | --- | --- | --- | --- | --- | --- |
| **Variables** | **Training group, n=329** | | |  | **Validation group, n=150** | | |
|  | **N** | **%** | **Median Overall Survival (95%CI), mo** |  | **N** | **%** | **Median Overall Survival (95%CI), mo** |
| **Age at diagnosis, Y** | | |  |  |  |  |  |
| <65 | 146 | 44.4 | 60.4 (15.8-NA) |  | 64 | 42.7 | 73.2 (57.2-89.2)# |
| ≥65 | 183 | 55.6 | 25.9 (19.8-32.2) |  | 86 | 57.3 | 26.4 (7.5-50.7) |
| **Sex** |  |  |  |  |  |  |  |
| Male | 212 | 64.4 | 36.5 (17.8-NA) |  | 93 | 62.0 | 29.1 (7.6-50.7) |
| Female | 117 | 35.6 | 29.0 (14.4-43.5) |  | 57 | 38.0 | 26.8 (NA) |
| **Primary site** |  |  |  |  |  |  |  |
| Cardia | 78 | 23.7 | 26.1 (17.2-34.9) |  | 35 | 23.3 | 22.5 (12.6-32.4) |
| Fundus**/**Body | 118 | 35.9 | 29.1 (15.0-43.3) |  | 62 | 41.3 | 28.9 (17.7-40.2) |
| Antrum | 126 | 38.3 | 38.4 (23.8-53.1) |  | 51 | 34.0 | 32.4 (9.2-67.3) |
| Unknown | 7 | 2.1 | 64.6 (25.1-84.2) |  | 2 | 1.3 | 42.5 (NA) |
| **Pathology grade** | | |  |  |  |  |  |
| I-II | 125 | 38.0 | 56.2 (32.4-80.0) |  | 58 | 38.7 | 58.2 (40.2-76.3) |
| III-IV | 195 | 59.3 | 26.8 (16.0-37.7) |  | 85 | 56.7 | 26.7 (19.8-33.6) |
| Unknown | 9 | 2.7 | 20.2 (10.6-41.1) |  | 7 | 4.7 | 20.2 (0-42.9) |
| **Lauren classification** | | |  |  |  |  |  |
| Intestinal type | 153 | 46.5 | 38.4 (15.1-61.8) |  | 70 | 22.0 | 58.2 (16.9-99.6) |
| Diffused type | 69 | 21.0 | 26.8 (5.7-48.0) |  | 33 | 46.7 | 26.8 (22.8-30.8) |
| Unknown | 107 | 32.5 | 26.7 (7.0-46.4) |  | 47 | 31.3 | 25.9 (11.9-39.9) |
| **T staging** |  |  |  |  |  |  |  |
| T1 | 17 | 5.2 | 58.2 (25.6-70.9)# |  | 9 | 6.0 | 52.8 (NA)# |
| T2 | 56 | 17.0 | 60.4 (2.6-118.1) |  | 26 | 17.3 | 26.0 (8.1-44.0) |
| T3 | 125 | 38.0 | 29.1 (NA) |  | 52 | 34.7 | 26.7 (25.6-27.8) |
| T4 | 131 | 39.8 | 27.7 (14.8-40.6) |  | 63 | 42.0 | 38.4 (7.5-69.3) |
| **N staging** |  |  |  |  |  |  |  |
| N0 | 100 | 30.4 | 60.4 (26.0-94.7) |  | 48 | 32.0 | 73.2 (NA) |
| N1 | 91 | 27.7 | 46.9 (18.9-74.9) |  | 37 | 24.7 | 58.2 (3.0-113.4) |
| N2 | 59 | 17.9 | 29.0 (16.8-41.1) |  | 24 | 16.0 | 29.0 (15.8-42.1) |
| N3 | 79 | 24.0 | 21.1 (12.6-29.6) |  | 41 | 27.3 | 22.5 (10.3-34.7) |
| **M staging** |  |  |  |  |  |  |  |
| M0 | 310 | 94.2 | 38.4 (20.2-56.7) |  | 141 | 94.0 | 38.4 (10.5-66.4) |
| M1 | 19 | 5.8 | 8.6 (4.9-12.4) |  | 9 | 6.0 | 8.6 (3.2-14.0) |
| **Pathological stage** | | |  |  |  |  |  |
| I | 38 | 11.6 | 73.2 (54.5-91.9) |  | 21 | 14.0 | 73.2 (NA) |
| II | 117 | 35.6 | 56.2 (23.7-88.7) |  | 46 | 30.7 | 31.2 (NA) |
| III | 155 | 47.1 | 26.5 (21.9-31.1) |  | 74 | 49.3 | 28.9 (16.3-41.6) |
| IV | 19 | 5.8 | 8.6 (4.9-12.4) |  | 9 | 6.0 | 8.6 (3.2-14.0) |
| **Surgery** |  |  |  |  |  |  |  |
| R0 | 280 | 85.1 | 43.1 (23.2-63.1) |  | 131 | 87.3 | 38.4 (12.4-64.5) |
| R1 | 14 | 4.3 | 17.1 (3.3-30.9) |  | 3 | 2.0 | 23.7 (0-51.0) |
| R2 | 9 | 2.7 | 9.9 (0-22.8) |  | 3 | 2.0 | 2.7 (2.7-2.8) |
| Unknown | 26 | 7.9 | 14.3 (10.0-18.5) |  | 13 | 8.7 | 14.3 (5.4-23.2) |
| **Chemotherapy** | |  |  |  |  |  |  |
| Yes | 146 | 44.4 | 34.8 (8.9-60.6) |  | 62 | 41.3 | 29.3 (13.8-44.9) |
| No | 183 | 55.6 | 26.5 (13.1-39.8) |  | 88 | 58.7 | 29.1 (0.2-58.1) |
| **Genomics score*** | | |  |  |  |  |  |
| Low | 165 | 50.2 | 87.1 (73.6-100.6)# |  | 65 | 43.3 | 88.8 (65.9-111.6)# |
| High | 164 | 49.8 | 15.5 (12.4-18.8) |  | 85 | 56.7 | 14.3 (9.7-18.8) |

*****based on Genomics nomogram

#based on average value

| **Table S2. Multivariable analysis for miRNAs** | | | | | | |
| --- | --- | --- | --- | --- | --- | --- |
| **ID** | **Coefficient** | **HR** | **95% CI** | **SE** | **Wald** | **p value** |
| **hsa-mir-100** | 0.155 | 1.167 | 0.967-1.408 | 0.967 | 2.608 | 0.106 |
| **hsa-mir-1304** | 0.149 | 1.161 | 1.039-1.298 | 1.039 | 6.925 | 0.008 |
| **hsa-mir-193b** | 0.130 | 1.139 | 0.964-1.346 | 0.964 | 2.338 | 0.126 |
| **hsa-mir-22** | 0.065 | 1.067 | 0.857-1.330 | 0.857 | 0.337 | 0.561 |
| **hsa-mir-136** | 0.074 | 1.077 | 0.886-1.309 | 0.886 | 0.555 | 0.456 |
| **hsa-mir-653** | 0.128 | 1.137 | 1.004-1.288 | 1.004 | 4.068 | 0.044 |
| **hsa-mir-6808** | 0.107 | 1.113 | 0.942-1.315 | 0.942 | 1.595 | 0.207 |

| **Table S3. Multivariable analysis for mRNA** | | | | | | |
| --- | --- | --- | --- | --- | --- | --- |
| **ID** | **Coefficient** | **HR** | **95% CI** | **SE** | **Wald** | **p value** |
| **NRP1\|8829** | -0.065 | 0.937 | 0.769-1.143 | 0.769 | 0.410 | 0.522 |
| **SOX14\|8403** | -0.381 | 0.683 | 0.512-0.913 | 0.512 | 6.632 | 0.010 |
| **CPNE8\|144402** | 0.208 | 1.231 | 1.024-1.408 | 1.024 | 4.880 | 0.027 |
| **MAGED1\|9500** | 0.302 | 1.353 | 1.137-1.610 | 1.137 | 11.585 | 0.001 |
| **RNF144A\|9781** | 0.140 | 1.150 | 0.933-1.419 | 0.933 | 1.711 | 0.191 |
| **ZNF22\|7570** | 0.241 | 1.273 | 1.076-1.505 | 1.076 | 7.965 | 0.005 |
| **DUSP1\|1843** | 0.240 | 1.272 | 1.060-1.526 | 1.060 | 6.677 | 0.010 |
| **LOC91450\|91450** | 0.227 | 1.254 | 1.086-1.449 | 1.086 | 9.469 | 0.002 |

| **Table S4. Multivariable analysis for DNA methylation site** | | | | | | |
| --- | --- | --- | --- | --- | --- | --- |
| **ID** | **Coefficient** | **HR** | **95% CI** | **SE** | **Wald** | **p value** |
| **cg02223323** | -.0280 | 0.972 | 0.797-1.186 | 0.101 | 0.078 | 0.780 |
| **cg00481239** | -0.354 | 0.702 | 0.435-1.133 | 0.244 | 2.098 | 0.147 |
| **cg07020967** | 0.354 | 1.425 | 1.118-1.816 | 0.124 | 8.214 | 0.004 |
| **cg08859156** | 0.306 | 1.358 | 1.143-1.613 | 0.088 | 12.105 | 0.001 |
| **cg12485556** | 0.504 | 1.655 | 1.205-2.274 | 0.162 | 9.680 | 0.002 |
| **cg14791193** | -0.114 | 0.893 | 0.739-1.079 | 0.097 | 1.382 | 0.240 |
| **cg15861578** | 0.233 | 1.263 | 1.020-1.563 | 0.109 | 4.581 | 0.032 |
| **cg15486740** | -0.354 | 0.702 | 0.563-0.874 | 0.112 | 9.967 | 0.002 |
| **cg20100408** | -0.320 | 0.726 | 0.603-0.873 | 0.094 | 11.512 | 0.001 |
| **cg20350671** | -0.178 | 0.837 | 0.677-1.036 | 0.109 | 2.679 | 0.102 |
| **cg22395807** | -0.421 | 0.656 | 0.444-0.970 | 0.199 | 4.471 | 0.034 |
| **cg24361571** | -0.102 | 0.903 | 0.724-1.126 | 0.113 | 0.819 | 0.365 |
| **cg25361506** | -0.195 | 0.822 | 0.668-1.013 | 0.106 | 3.393 | 0.065 |
| **cg25622155** | 0.155 | 1.168 | 0.977-1.397 | 0.091 | 2.910 | 0.088 |
| **cg25161386** | 0.022 | 1.023 | 0.823-1.270 | 0.111 | 0.041 | 0.840 |
| **cg22740006** | 0.294 | 1.342 | 1.064-1.692 | 0.118 | 6.183 | 0.013 |
| **cg22813794** | -0.353 | 0.703 | 0.507-0.974 | 0.167 | 4.480 | 0.034 |
| **cg26014401** | -0.157 | 0.855 | 0.684-1.068 | 0.114 | 1.902 | 0.168 |
| **cg26856948** | -0.139 | 0.871 | 0.723-1.048 | 0.095 | 2.139 | 0.144 |

| **Table S5. Multivariable analysis for miRNAs, mRNA and DNA methylation and clinical characteristics** | | | | | | |
| --- | --- | --- | --- | --- | --- | --- |
| **ID** | **Coefficient** | **HR** | **95% CI** | **SE** | **Wald** | **p value** |
| **hsa-mir-100** | 0.089 | 1.093 | 0.820-1.456 | 0.146 | 0.367 | 0.544 |
| **hsa-mir-1304** | -0.022 | .979 | 0.821-1.166 | 0.089 | 0.059 | 0.808 |
| **hsa-mir-193b** | 0.204 | 1.226 | 1.003-1.498 | 0.102 | 3.963 | 0.047 |
| **hsa-mir-22** | -0.263 | .769 | 0.534-1.107 | 0.186 | 2.001 | 0.157 |
| **hsa-mir-136** | -0.044 | .957 | 0.714-1.283 | 0.149 | 0.086 | 0.770 |
| **hsa-mir-653** | -0.093 | .911 | 0.775-1.071 | 0.082 | 1.265 | 0.261 |
| **hsa-mir-6808** | 0.003 | 1.003 | 0.815-1.236 | 0.106 | 0.001 | 0.975 |
| **NRP1\|8829** | -0.050 | 0.951 | 0.721-1.255 | 0.142 | 0.125 | 0.723 |
| **SOX14\|8403** | -0.313 | 0.731 | 0.516-1.037 | 0.178 | 3.078 | 0.079 |
| **CPNE8\|144402** | -0.015 | 0.985 | 0.780-1.244 | 0.119 | 0.016 | 0.898 |
| **MAGED1\|9500** | -0.033 | 0.968 | 0.737-1.271 | 0.139 | 0.055 | 0.815 |
| **RNF144A\|9781** | -0.055 | 0.947 | 0.742-1.209 | 0.125 | 0.193 | 0.661 |
| **ZNF22\|7570** | 0.317 | 1.373 | 1.077-1.751 | 0.124 | 6.531 | 0.011 |
| **DUSP1\|1843** | 0.146 | 1.157 | 0.945-1.417 | 0.103 | 1.990 | 0.158 |
| **LOC91450\|91450** | -0.045 | 0.956 | 0.761-1.202 | 0.117 | 0.147 | 0.701 |
| **cg02223323** | -0.169 | 0.844 | 0.652-1.093 | 0.132 | 1.653 | 0.199 |
| **cg00481239** | -0.350 | 0.705 | 0.409-1.213 | 0.277 | 1.596 | 0.207 |
| **cg07020967** | 0.492 | 1.635 | 1.192-2.243 | 0.161 | 9.296 | 0.002 |
| **cg08859156** | 0.190 | 1.209 | 0.999-1.462 | 0.097 | 3.821 | 0.051 |
| **cg12485556** | 0.243 | 1.275 | 0.870-1.867 | 0.195 | 1.550 | 0.213 |
| **cg14791193** | -0.091 | 0.913 | 0.723-1.152 | 0.119 | 0.593 | 0.441 |
| **cg15861578** | 0.338 | 1.402 | 1.086-1.811 | 0.130 | 6.720 | 0.010 |
| **cg15486740** | -0.544 | 0.581 | 0.445-0.757 | 0.135 | 16.136 | <0.001 |
| **cg20100408** | -0.238 | 0.788 | 0.622-0.998 | 0.121 | 3.895 | 0.048 |
| **cg20350671** | -0.322 | 0.725 | 0.549-0.958 | 0.142 | 5.105 | 0.024 |
| **cg22395807** | -0.299 | 0.742 | 0.514-1.070 | 0.187 | 2.559 | 0.110 |
| **cg24361571** | -0.199 | 0.819 | 0.632-1.063 | 0.133 | 2.254 | 0.133 |
| **cg25361506** | -0.154 | 0.857 | 0.673-1.092 | 0.123 | 1.559 | 0.212 |
| **cg25622155** | 0.160 | 1.173 | 0.944-1.459 | 0.111 | 2.067 | 0.151 |
| **cg25161386** | -0.013 | 0.987 | 0.750-1.298 | 0.140 | 0.009 | 0.924 |
| **cg22740006** | 0.286 | 1.331 | 1.026-1.726 | 0.133 | 4.649 | 0.031 |
| **cg22813794** | -0.401 | 0.670 | 0.466-0.962 | 0.185 | 4.706 | 0.030 |
| **cg26014401** | -0.090 | 0.914 | 0.701-1.191 | 0.135 | 0.446 | 0.504 |
| **cg26856948** | -0.230 | 0.795 | 0.631-1.001 | 0.118 | 3.794 | 0.051 |
| **year** | -0.006 | 0.994 | 0.908-1.089 | 0.047 | 0.014 | 0.905 |
| **age** | 0.035 | 1.035 | 1.012-1.059 | 0.012 | 9.061 | 0.003 |
| **sex** | -0.273 | 0.761 | 0.440-1.316 | 0.279 | 0.955 | 0.329 |
| **site** | -0.054 | 0.947 | 0.713-1.259 | 0.145 | 0.140 | 0.708 |
| **lauren** | 0.111 | 1.118 | 0.833-1.499 | 0.150 | 0.550 | 0.458 |
| **grade** | 0.306 | 1.358 | 0.946-1.951 | 0.185 | 2.746 | 0.097 |
| **T** | 0.370 | 1.448 | 1.069-1.961 | 0.155 | 5.719 | 0.017 |
| **N** | 0.102 | 1.107 | 0.922-1.329 | 0.093 | 1.193 | 0.275 |
| **M** | 1.209 | 3.352 | 1.508-7.451 | 0.408 | 8.805 | 0.003 |
| **surgery** | 0.299 | 1.349 | 1.069-1.702 | 0.119 | 6.376 | 0.012 |
| **target therapy** | -0.160 | 0.852 | 0.721-1.006 | 0.085 | 3.557 | 0.059 |
| **chemotherapy** | 0.009 | 1.009 | 0.540-1.886 | 0.319 | 0.001 | 0.978 |

| **Table S6. The** **equation of different models** | |
| --- | --- |
| **Models** | **Equation** |
| **Genomics nomogram** | 0.234*hsa-mir-100+0.113*hsa-mir-1304+0.235*hsa-mir-136+0.241*hsa-mir-193b+0.248*hsa-mir-22+0.148*hsa-mir-653+0.180*hsa-mir-6808+0.291*NRP1\|8829+0.313*RNF144A\|9781+0.302*ZNF22\|7570+(-0.464)*SOX14\|8403+0.360*DUSP1\|1843+0.342*CPNE8\|144402+0.291*MAGED1\|9500+0.278*LOC91450\|91450+(-0.360)*cg02223323+(-0.668)*cg00481239+0.380*cg07020967+0.409*cg08859156+0.435*cg12485556+(-0.400)*cg14791193+0.329*cg15861578+(-0.363)*cg15486740+(-0.357)*cg20100408+(-0.390)*cg20350671+(-0.443)*cg22395807+(-0.340)*cg24361571+(-0.362)*cg25361506+(0.331)*cg25622155+0.305*cg25161386+0.342*cg22740006+(-0.351)*cg22813794+(-0.430)*cg26014401+(-0.334)*cg26856948 |
| **miRNAs nomogram** | 0.234*hsa-mir-100+0.113*hsa-mir-1304+0.235*hsa-mir-136+0.241*hsa-mir-193b+0.248*hsa-mir-22+0.148*hsa-mir-653+0.180*hsa-mir-6808 |
| **mRNA nomogram** | 0.291*NRP1\|8829+0.313*RNF144A\|9781+0.302*ZNF22\|7570+(-0.464)*SOX14\|8403+0.360*DUSP1\|1843+0.342*CPNE8\|144402+0.291*MAGED1\|9500+0.278*LOC91450\|91450 |
| **Metylation nomogram** | (-0.360)*cg02223323+(-0.668)*cg00481239+0.380*cg07020967+0.409*cg08859156+0.435*cg12485556+(-0.400)*cg14791193+0.329*cg15861578+(-0.363)*cg15486740+(-0.357)*cg20100408+(-0.390)*cg20350671+(-0.443)*cg22395807+(-0.340)*cg24361571+(-0.362)*cg25361506+(0.331)*cg25622155+0.305*cg25161386+0.342*cg22740006+(-0.351)*cg22813794+(-0.430)*cg26014401+(-0.334)*cg26856948 |
| **miRNAs+Metylation nomogram** | 0.234*hsa-mir-100+0.113*hsa-mir-1304+0.235*hsa-mir-136+0.241*hsa-mir-193b+0.248*hsa-mir-22+0.148*hsa-mir-653+0.180*hsa-mir-6808+(-0.360)*cg02223323+(-0.668)*cg00481239+0.380*cg07020967+0.409*cg08859156+0.435*cg12485556+(-0.400)*cg14791193+0.329*cg15861578+(-0.363)*cg15486740+(-0.357)*cg20100408+(-0.390)*cg20350671+(-0.443)*cg22395807+(-0.340)*cg24361571+(-0.362)*cg25361506+(0.331)*cg25622155+0.305*cg25161386+0.342*cg22740006+(-0.351)*cg22813794+(-0.430)*cg26014401+(-0.334)*cg26856948 |
| **miRNAs+mRNA nomogram** | 0.234*hsa-mir-100+0.113*hsa-mir-1304+0.235*hsa-mir-136+0.241*hsa-mir-193b+0.248*hsa-mir-22+0.148*hsa-mir-653+0.180*hsa-mir-6808+0.291*NRP1\|8829+0.313*RNF144A\|9781+0.302*ZNF22\|7570+(-0.464)*SOX14\|8403+0.360*DUSP1\|1843+0.342*CPNE8\|144402+0.291*MAGED1\|9500+0.278*LOC91450\|91450 |
| **mRNA+Metylation nomogram** | 0.291*NRP1\|8829+0.313*RNF144A\|9781+0.302*ZNF22\|7570+(-0.464)*SOX14\|8403+0.360*DUSP1\|1843+0.342*CPNE8\|144402+0.291*MAGED1\|9500+0.278*LOC91450\|91450+(-0.360)*cg02223323+(-0.668)*cg00481239+0.380*cg07020967+0.409*cg08859156+0.435*cg12485556+(-0.400)*cg14791193+0.329*cg15861578+(-0.363)*cg15486740+(-0.357)*cg20100408+(-0.390)*cg20350671+(-0.443)*cg22395807+(-0.340)*cg24361571+(-0.362)*cg25361506+(0.331)*cg25622155+0.305*cg25161386+0.342*cg22740006+(-0.351)*cg22813794+(-0.430)*cg26014401+(-0.334)*cg26856948 |
| **Cox-model 1 nomogram** | 0.149*hsa-mir-1304+0.128*hsa-mir-653+0.313*RNF144A\|9781+(-0.381)*SOX14\|8403+0.240*DUSP1\|1843+0.208*CPNE8\|144402+0.302*MAGED1\|9500+0.227*LOC91450\|91450+0.354*cg07020967+0.306*cg08859156+0.504*cg12485556+0.233*cg15861578+(-0.354)*cg15486740+(-0.320)*cg20100408+(-0.421)*cg22395807+0.294*cg22740006+(-0.353)*cg22813794 |
| **Cox-model 2 nomogram** | 0.204*hsa-mir-193b+0.317*ZNF22\|7570+0.492*cg07020967+0.338*cg15861578+(-0.544)*cg15486740+(-0.238)*cg20100408+(-0.322)+cg20350671+0.286*cg22740006+(-0.401)*cg22813794 |

| **Table S7. The C-index value of different models** | | | | | | |
| --- | --- | --- | --- | --- | --- | --- |
| **Training group** | **C-index** | **DXY** | **SD** | **Z** | **P** | **n** |
| Genomics nomogram | 0.807 | 0.613 | 0.035 | 17.42 | <0.001 | 329 |
| Clinical nomogram | 0.629 | 0.259 | 0.005 | 5.21 | <0.001 | 329 |
| miRNAs nomogram | 0.676 | 0.352 | 0.049 | 7.16 | <0.001 | 329 |
| mRNA nomogram | 0.753 | 0.506 | 0.041 | 12.49 | <0.001 | 329 |
| Metylation nomogram | 0.795 | 0.590 | 0.036 | 16.17 | <0.001 | 329 |
| miRNAs+Metylation nomogram | 0.793 | 0.586 | 0.037 | 15.69 | <0.001 | 329 |
| miRNAs+mRNA nomogram | 0.745 | 0.490 | 0.041 | 11.88 | <0.001 | 329 |
| mRNA+Metylation nomogram | 0.805 | 0.611 | 0.034 | 17.20 | <0.001 | 329 |
| Cox-model 1 nomogram | 0.824 | 0.648 | 0.034 | 19.21 | <0.001 | 329 |
| Cox-model 2 nomogram | 0.802 | 0.602 | 0.035 | 16.89 | <0.001 | 329 |
| **Validation group** | **C-index** | **DXY** | **SD** | **Z** | **P** | **n** |
| Genomics nomogram | 0.823 | 0.645 | 0.054 | 11.94 | <0.001 | 150 |
| Clinical nomogram | 0.636 | 0.272 | 0.086 | 3.16 | <0.001 | 150 |
| miRNAs nomogram | 0.701 | 0.402 | 0.071 | 5.67 | <0.001 | 150 |
| mRNA nomogram | 0.782 | 0.564 | 0.059 | 9.64 | <0.001 | 150 |
| Metylation nomogram | 0.821 | 0.643 | 0.052 | 12.37 | <0.001 | 150 |
| miRNAs+Metylation nomogram | 0.823 | 0.646 | 0.051 | 12.56 | <0.001 | 150 |
| miRNAs+mRNA nomogram | 0.782 | 0.564 | 0.057 | 9.82 | <0.001 | 150 |
| mRNA+Metylation nomogram | 0.829 | 0.658 | 0.053 | 12.35 | <0.001 | 150 |
| Cox-model 1 nomogram | 0.841 | 0.682 | 0.049 | 13.87 | <0.001 | 150 |
| Cox-model 2 nomogram | 0.835 | 0.670 | 0.049 | 13.76 | <0.001 | 150 |

| **Table S8. The AUC values for each individual of genome-wide network** | | | | | |  |  |
| --- | --- | --- | --- | --- | --- | --- | --- |
| **Models** | **Training group, n=329** | | | **Validation group, n=150** | | | |
|  | **1-year OS** | **3-year OS** | **5-year OS** | **1-year OS** | **3-year OS** | **5-year OS** |  |
|  | **AUC** | **AUC** | **AUC** | **AUC** | **AUC** | **AUC** |  |
| **Genomics score** | 0.815 | 0.823 | 0.855 | 0.868 | 0.895 | 0.928 |  |
| **Clinical nomogram** | 0.609 | 0.615 | 0.642 | 0.638 | 0.598 | 0.721 |  |
| **hsa-mir-100** | 0.617 | 0.598 | 0.655 | 0.623 | 0.670 | 0.677 |  |
| **hsa-mir-1304** | 0.494 | 0.535 | 0.508 | 0.607 | 0.581 | 0.685 |  |
| **hsa-mir-193b** | 0.571 | 0.600 | 0.625 | 0.619 | 0.676 | 0.701 |  |
| **hsa-mir-22** | 0.613 | 0.610 | 0.695 | 0.599 | 0.657 | 0.685 |  |
| **hsa-mir-136** | 0.577 | 0.637 | 0.714 | 0.568 | 0.696 | 0.778 |  |
| **hsa-mir-653** | 0.620 | 0.599 | 0.781 | 0.584 | 0.625 | 0.792 |  |
| **hsa-mir-6808** | 0.540 | 0.609 | 0.600 | 0.615 | 0.687 | 0.806 |  |
| **NRP1\|8829** | 0.645 | 0.658 | 0.707 | 0.680 | 0.686 | 0.731 |  |
| **SOX14\|8403** | 0.525 | 0.516 | 0.550 | 0.560 | 0.607 | 0.597 |  |
| **CPNE8\|144402** | 0.656 | 0.618 | 0.602 | 0.727 | 0.686 | 0.701 |  |
| **MAGED1\|9500** | 0.621 | 0.590 | 0.513 | 0.653 | 0.626 | 0.657 |  |
| **RNF144A\|9781** | 0.598 | 0.588 | 0.682 | 0.602 | 0.536 | 0.614 |  |
| **ZNF22\|7570** | 0.618 | 0.596 | 0.590 | 0.583 | 0.597 | 0.653 |  |
| **DUSP1\|1843** | 0.626 | 0.563 | 0.513 | 0.637 | 0.574 | 0.574 |  |
| **LOC91450\|91450** | 0.614 | 0.621 | 0.610 | 0.527 | 0.603 | 0.516 |  |
| **cg02223323** | 0.645 | 0.594 | 0.606 | 0..619 | 0.651 | 0.736 |  |
| **cg00481239** | 0.576 | 0.558 | 0.635 | 0.562 | 0.580 | 0.570 |  |
| **cg07020967** | 0.647 | 0.612 | 0.536 | 0.694 | 0.671 | 0.611 |  |
| **cg08859156** | 0.583 | 0.561 | 0.680 | 0.607 | 0.575 | 0.796 |  |
| **cg12485556** | 0.584 | 0.667 | 0.647 | 0.542 | 0.647 | 0.535 |  |
| **cg14791193** | 0.654 | 0.556 | 0.580 | 0.728 | 0.549 | 0.652 |  |
| **cg15861578** | 0.571 | 0.652 | 0.680 | 0.520 | 0.667 | 0.656 |  |
| **cg15486740** | 0.660 | 0.609 | 0.573 | 0.763 | 0.660 | 0.567 |  |
| **cg20100408** | 0.629 | 0.663 | 0.635 | 0.611 | 0.609 | 0.665 |  |
| **cg20350671** | 0.636 | 0.576 | 0.642 | 0.645 | 0.542 | 0.591 |  |
| **cg22395807** | 0.556 | 0.561 | 0.538 | 0.563 | 0.599 | 0.555 |  |
| **cg24361571** | 0.632 | 0.587 | 0.621 | 0.620 | 0.620 | 0.564 |  |
| **cg25361506** | 0.612 | 0.658 | 0.810 | 0.681 | 0.651 | 0.879 |  |
| **cg25622155** | 0.635 | 0.619 | 0.686 | 0.616 | 0.667 | 0.709 |  |
| **cg25161386** | 0.579 | 0.619 | 0.642 | 0.607 | 0.678 | 0.668 |  |
| **cg22740006** | 0.575 | 0.636 | 0.602 | 0.635 | 0.707 | 0.656 |  |
| **cg22813794** | 0.504 | 0.551 | 0.663 | 0.517 | 0.535 | 0.535 |  |
| **cg26014401** | 0.623 | 0.652 | 0.540 | 0.743 | 0.696 | 0.639 |  |
| **cg26856948** | 0.652 | 0.570 | 0.550 | 0.650 | 0.580 | 0.593 |  |

II.


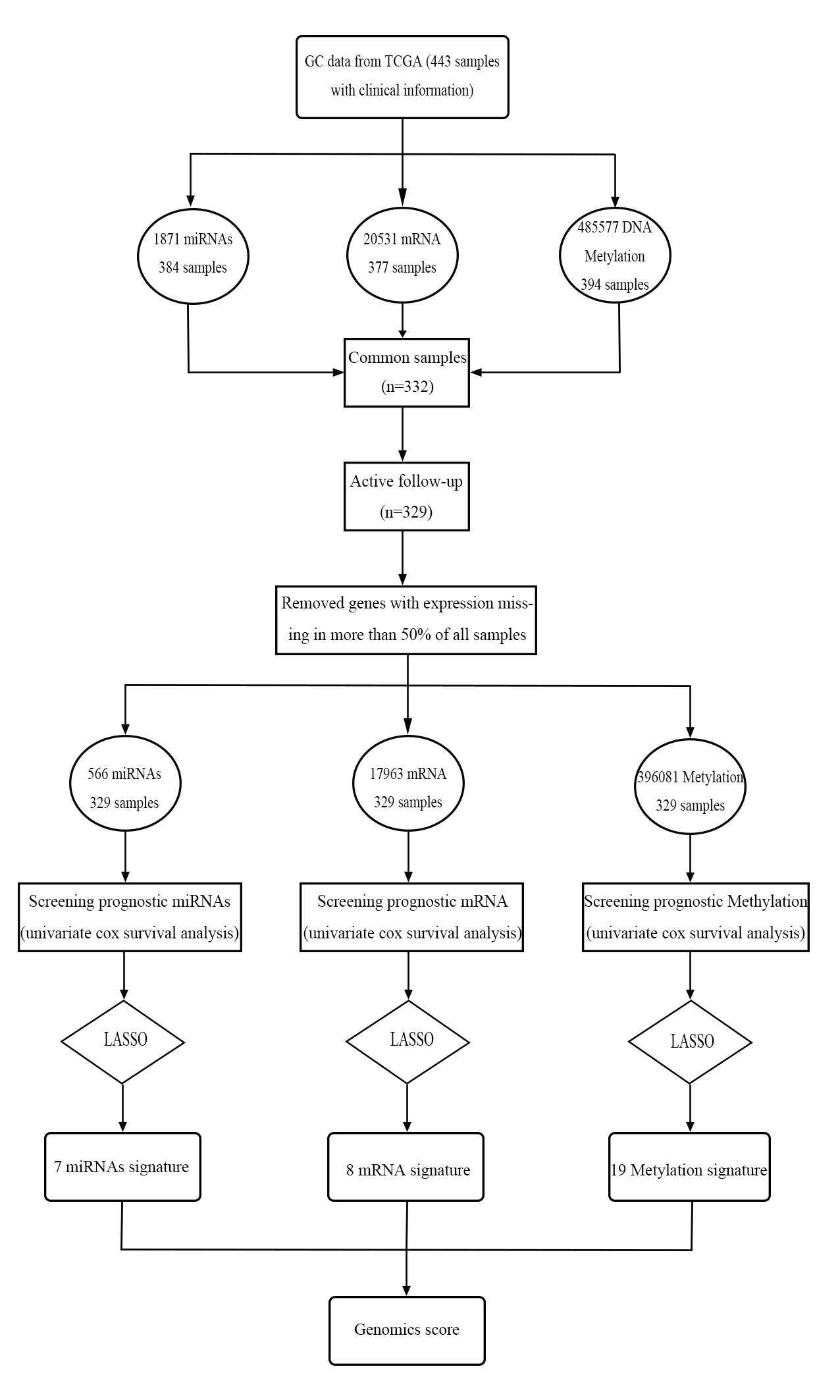


**Figure S1:** Screening process for genome-wide network


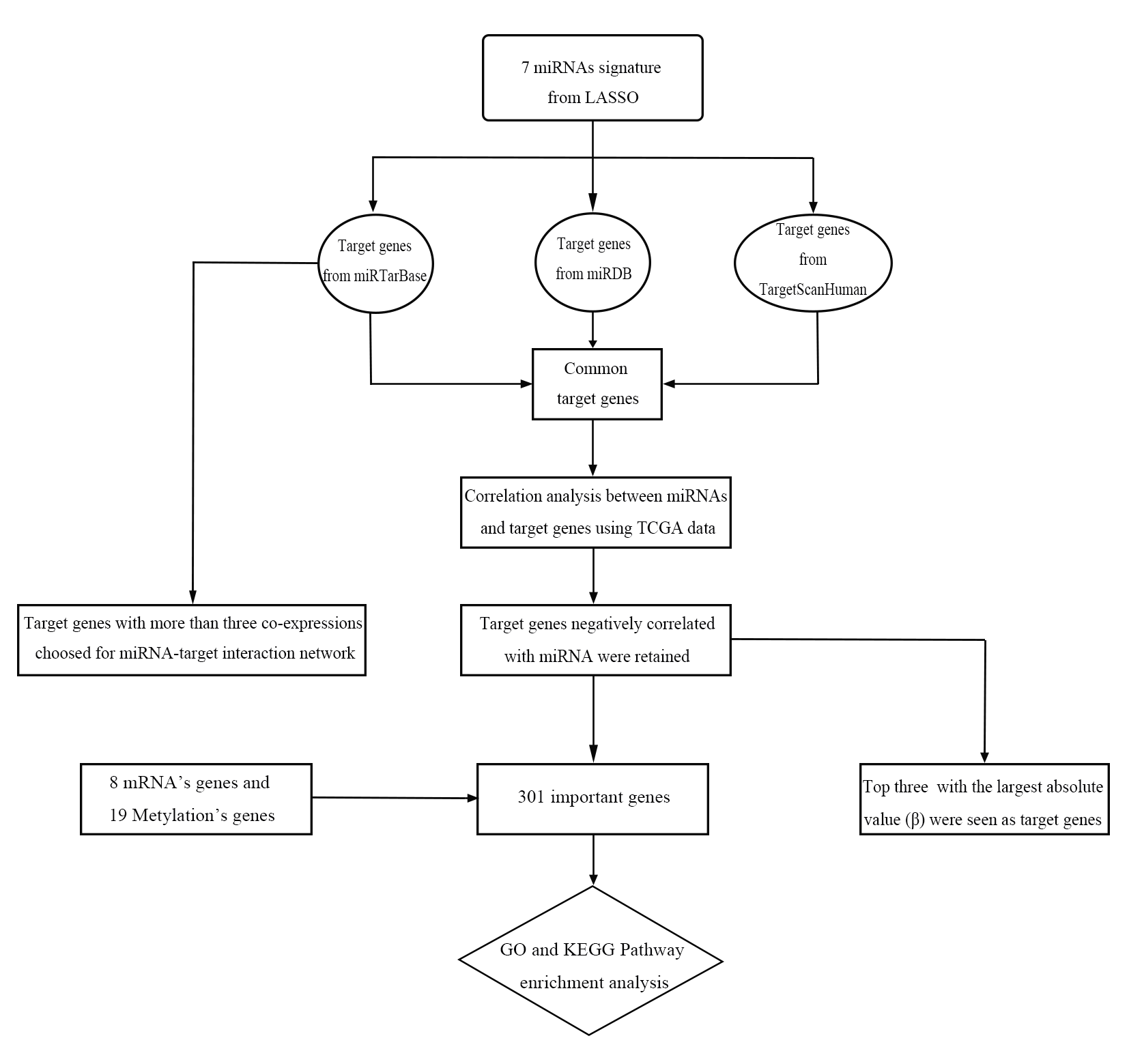


**Figure S2:** Screening process for GO analysis and KEGG pathways


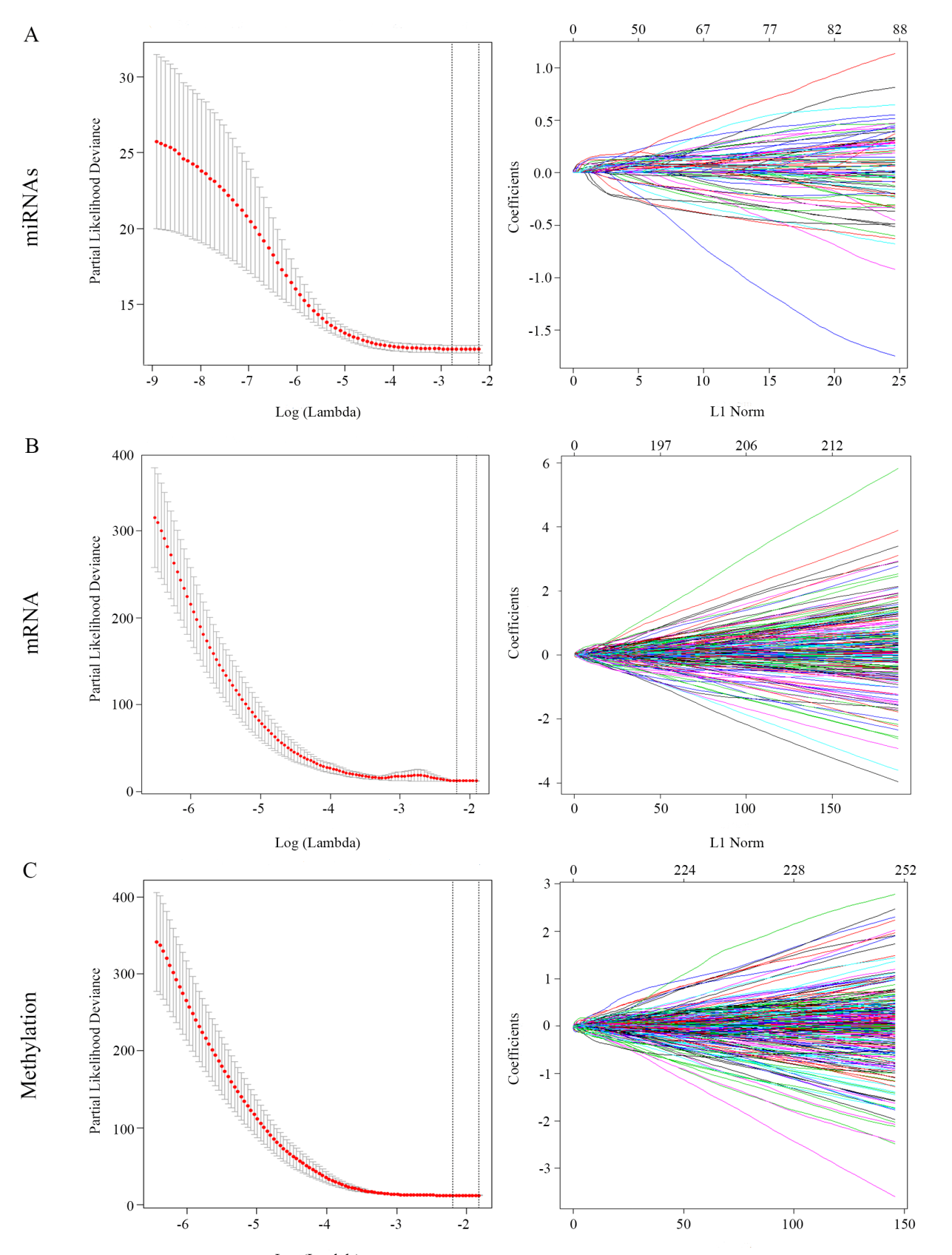


**Figure S3:** LASSO Cox regression performed for miRNAs, mRNA and DNA methylation sites


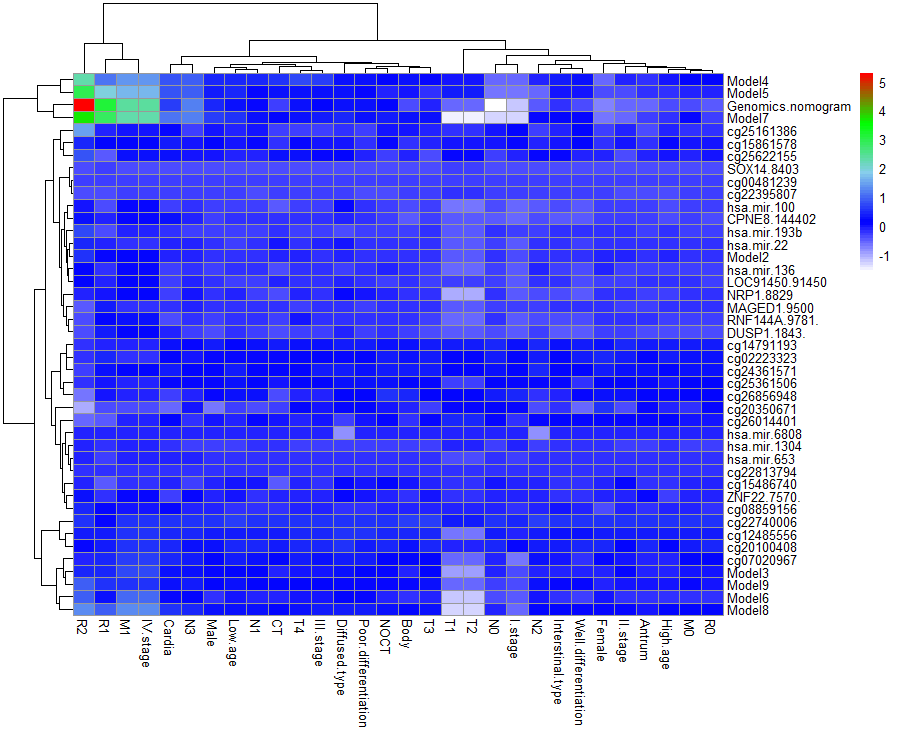


**Figure S4:** A heat map based on genomic scores layered by clinicopathological factors

**
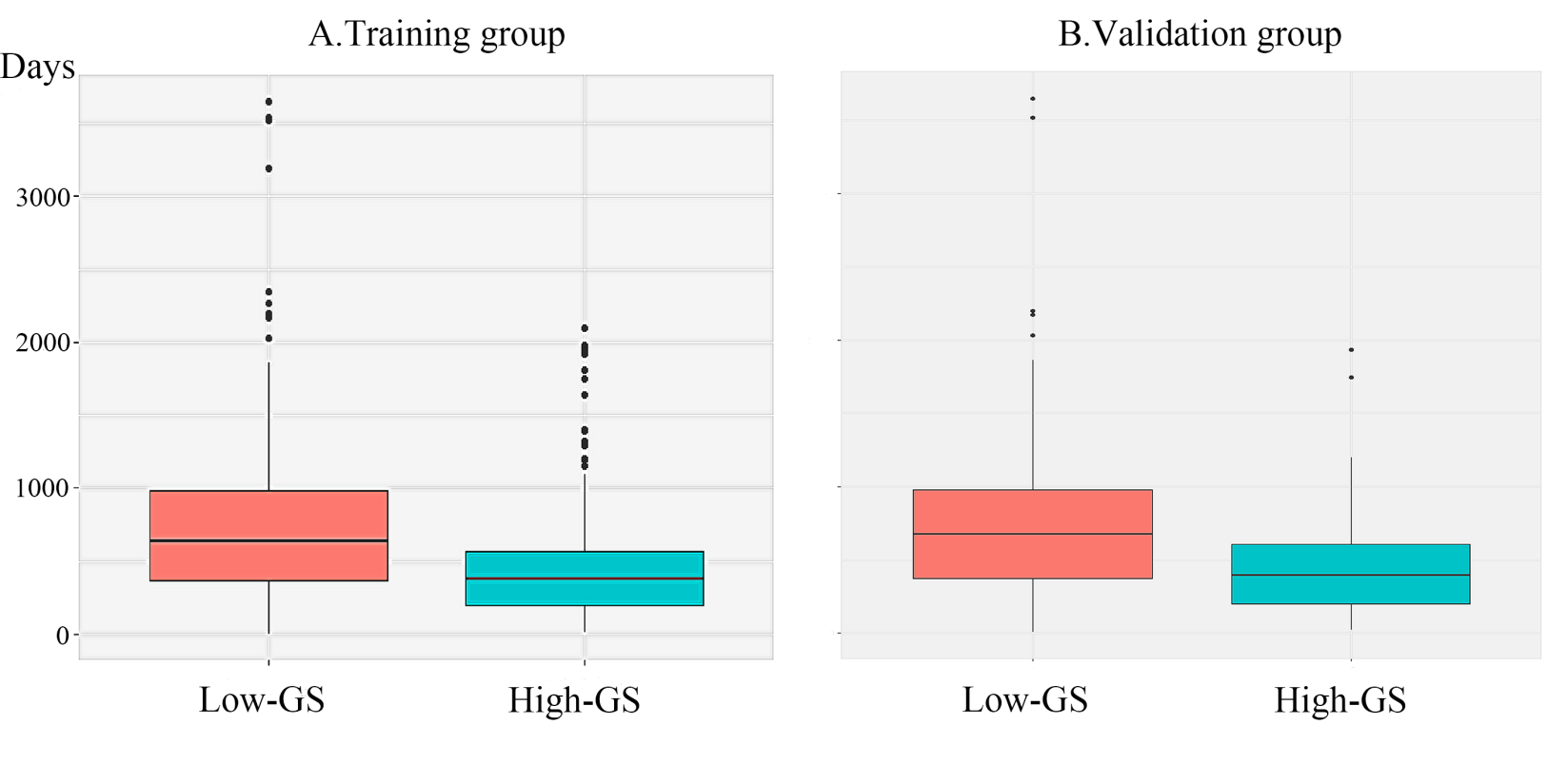
**

**Figure S5:** Median survival for patients in the low-GS and high-GS groups


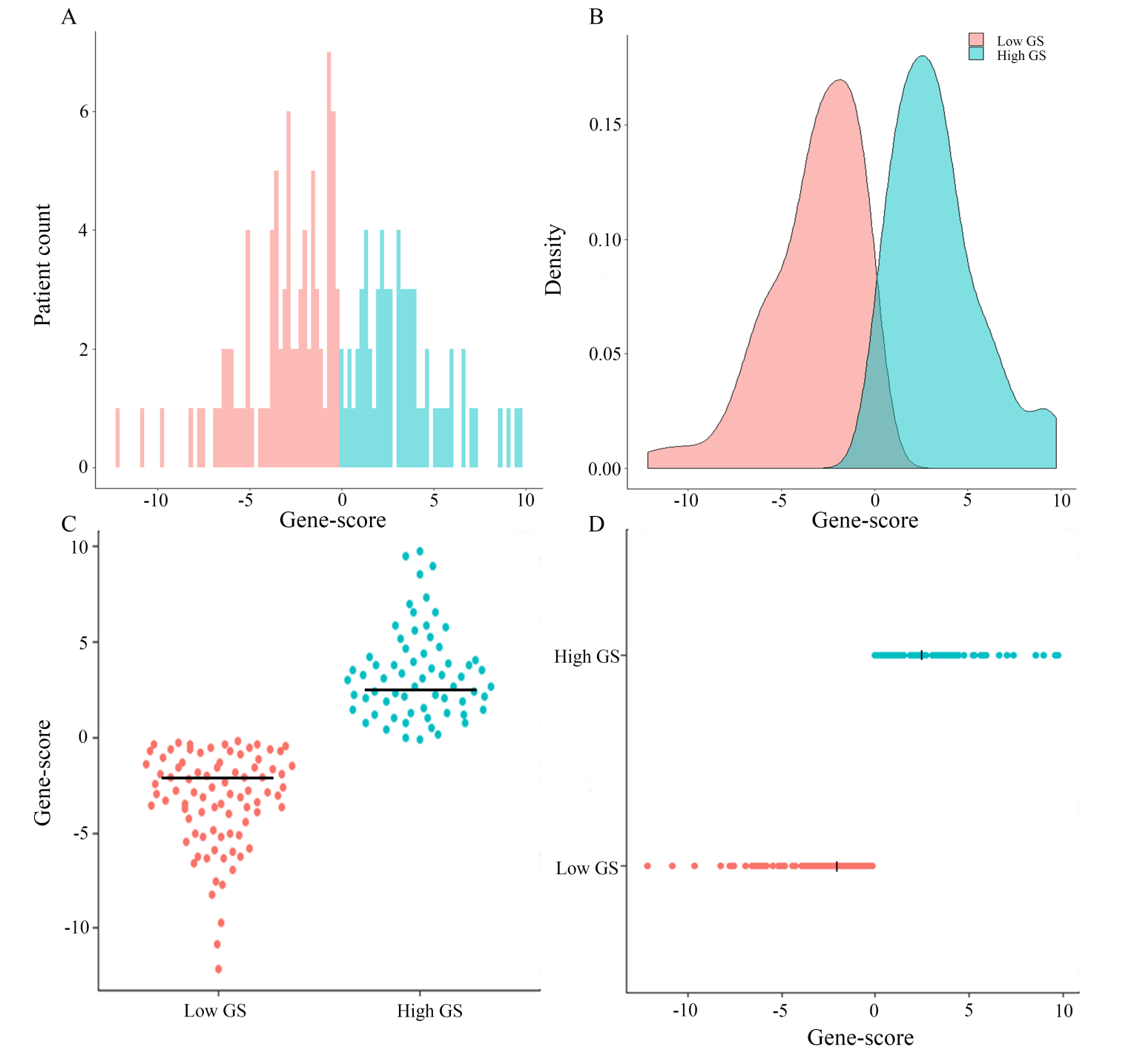


**Figure S6:** Distribution of patient cases and density based on genomics score in validation group (A and B), Scatter plots of genomics scores regarding the classification of low and high GS (C and D), and the bold line represents the median


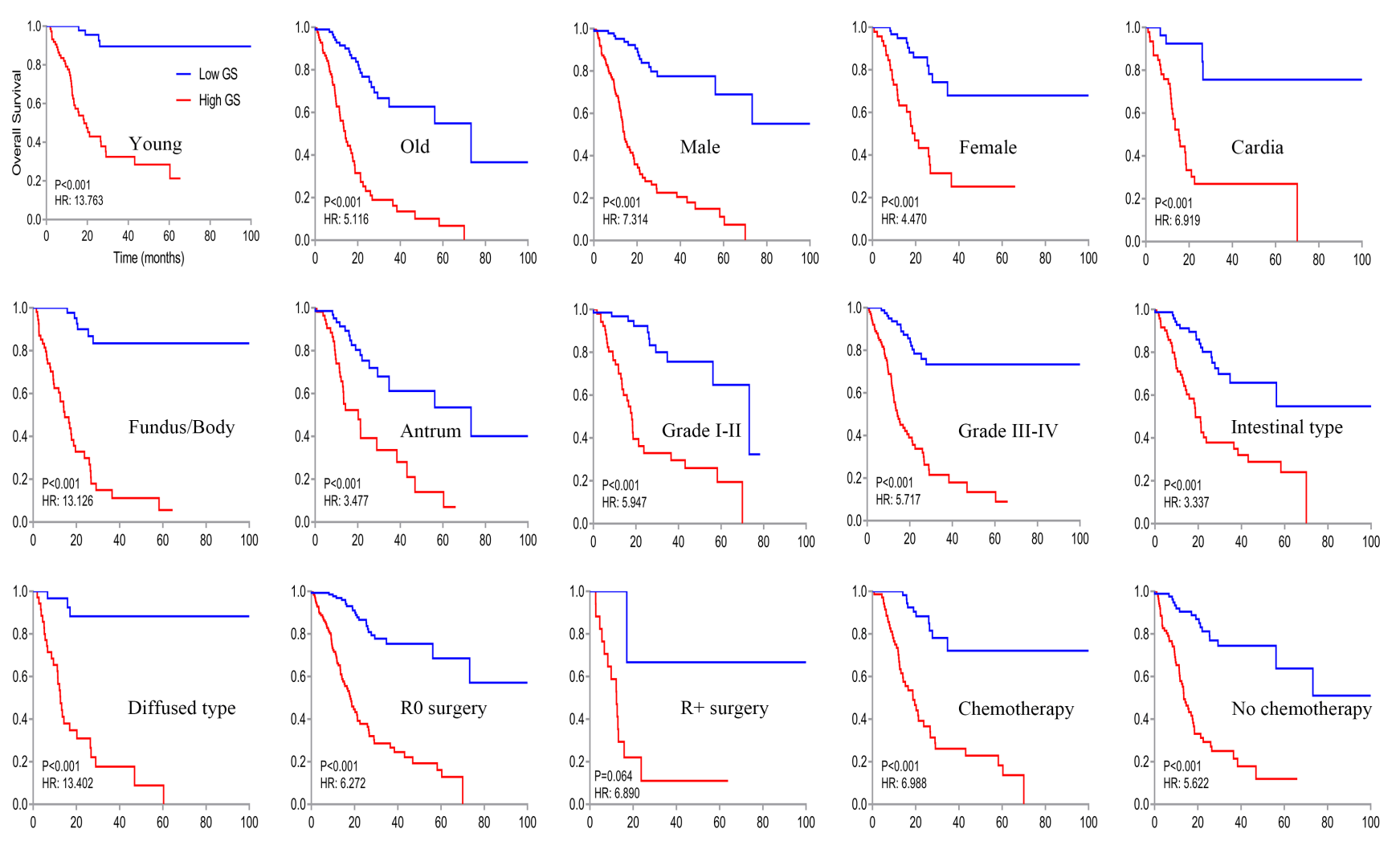


**Figure S7:**Kaplan-Meier curve of overall survival for the low- and high-GS groups divided by age (young and old), sex (male and female), primary site (cardia, fundus/body and antrum), pathology grade (I-II and III-IV), Lauren classification (intestinal type and diffused type) and treatment (R0 surgery, R1 surgery, chemotherapy and no chemotherapy) in training group


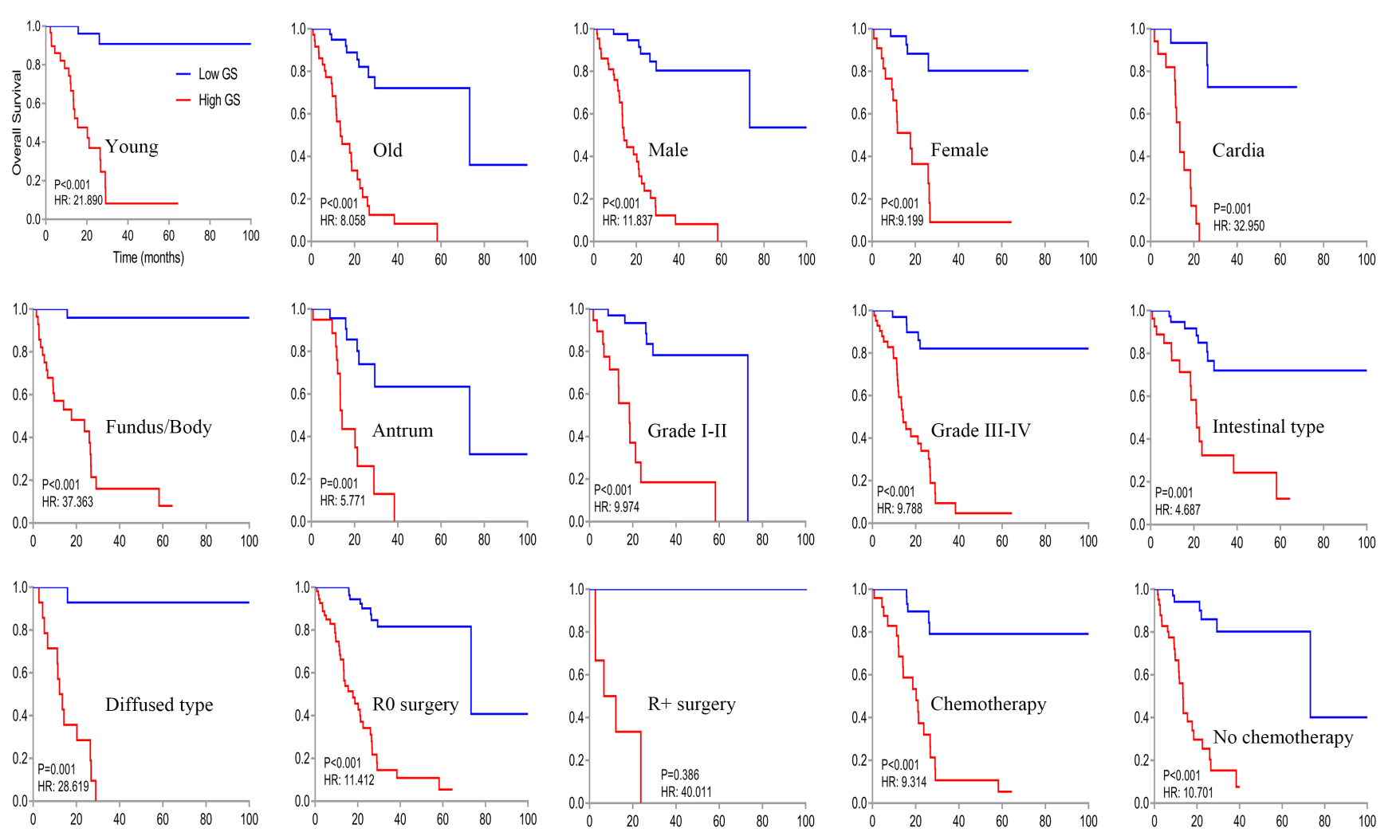


**Figure S8:** Kaplan-Meier curve of overall survival for the low- and high-GS groups divided by age (young and old), sex (male and female), primary site (cardia, fundus/body and antrum), pathology grade (I-II and III-IV), Lauren classification (intestinal type and diffused type) and treatment (R0 surgery, R1 surgery, chemotherapy and no chemotherapy) in validation group

**Figure S9:** Cox-model 1 nomogram and its calibration plot in training group and validation group
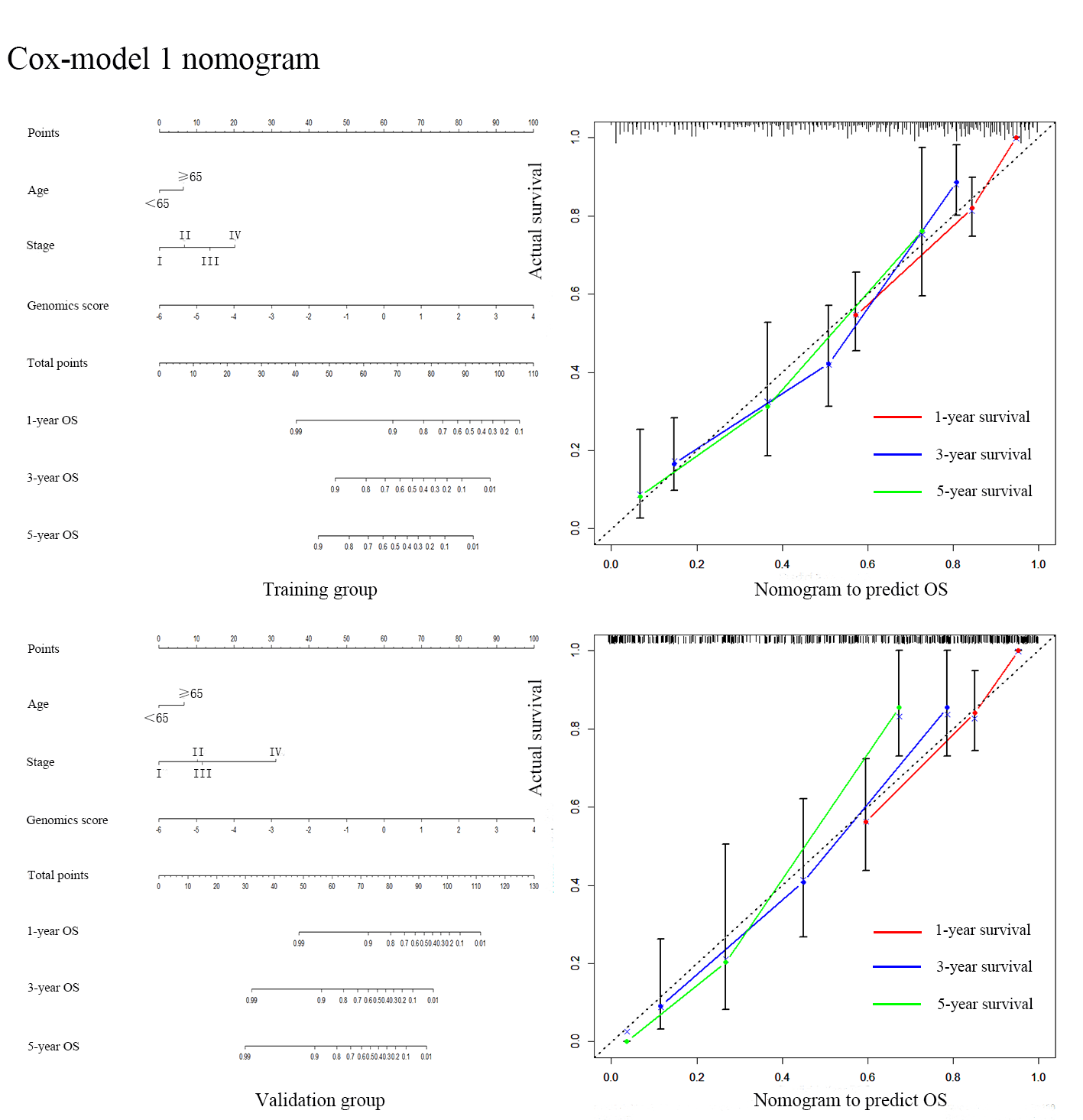


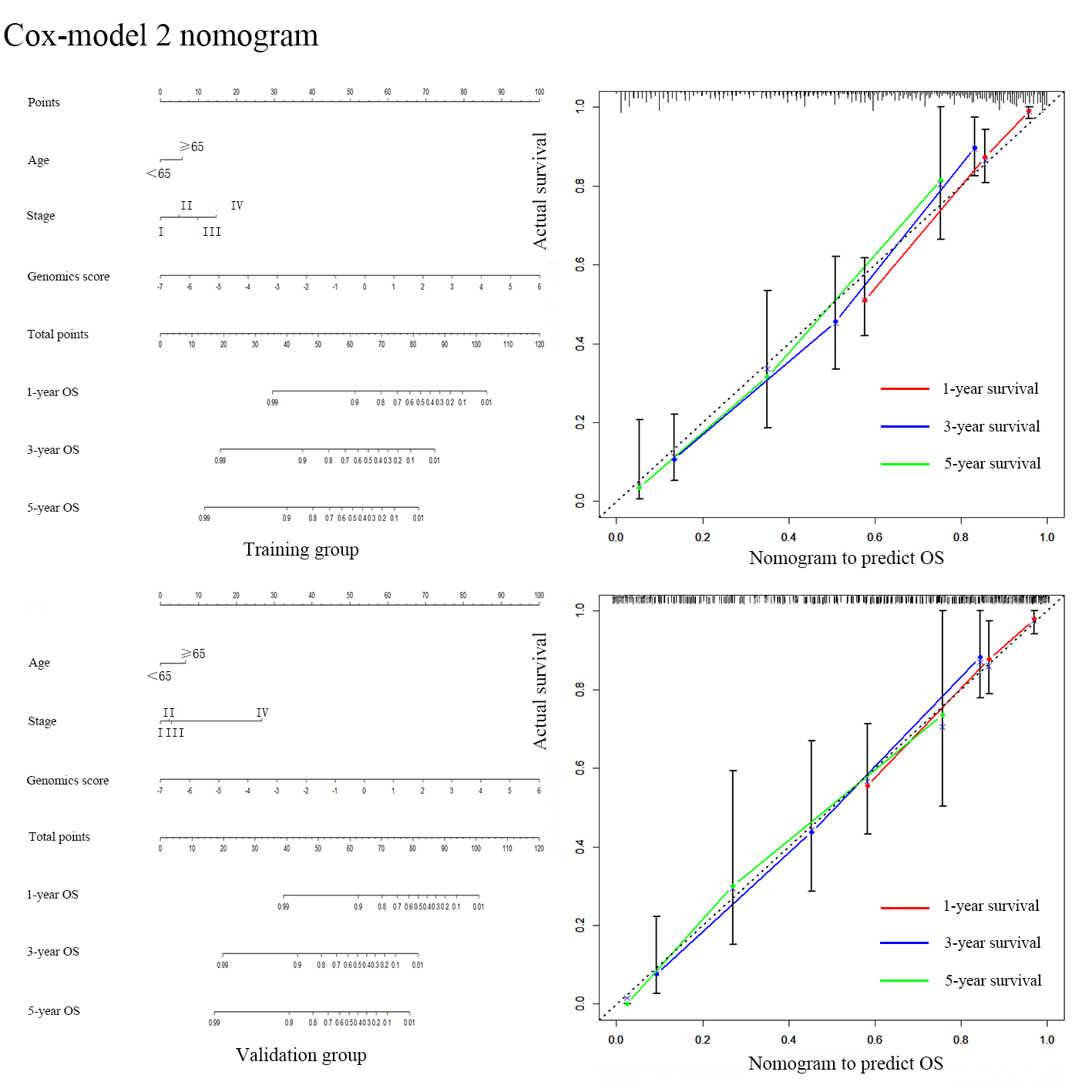


**Figure S10:** Cox-model 2 nomogram and its calibration plot in training group and validation group


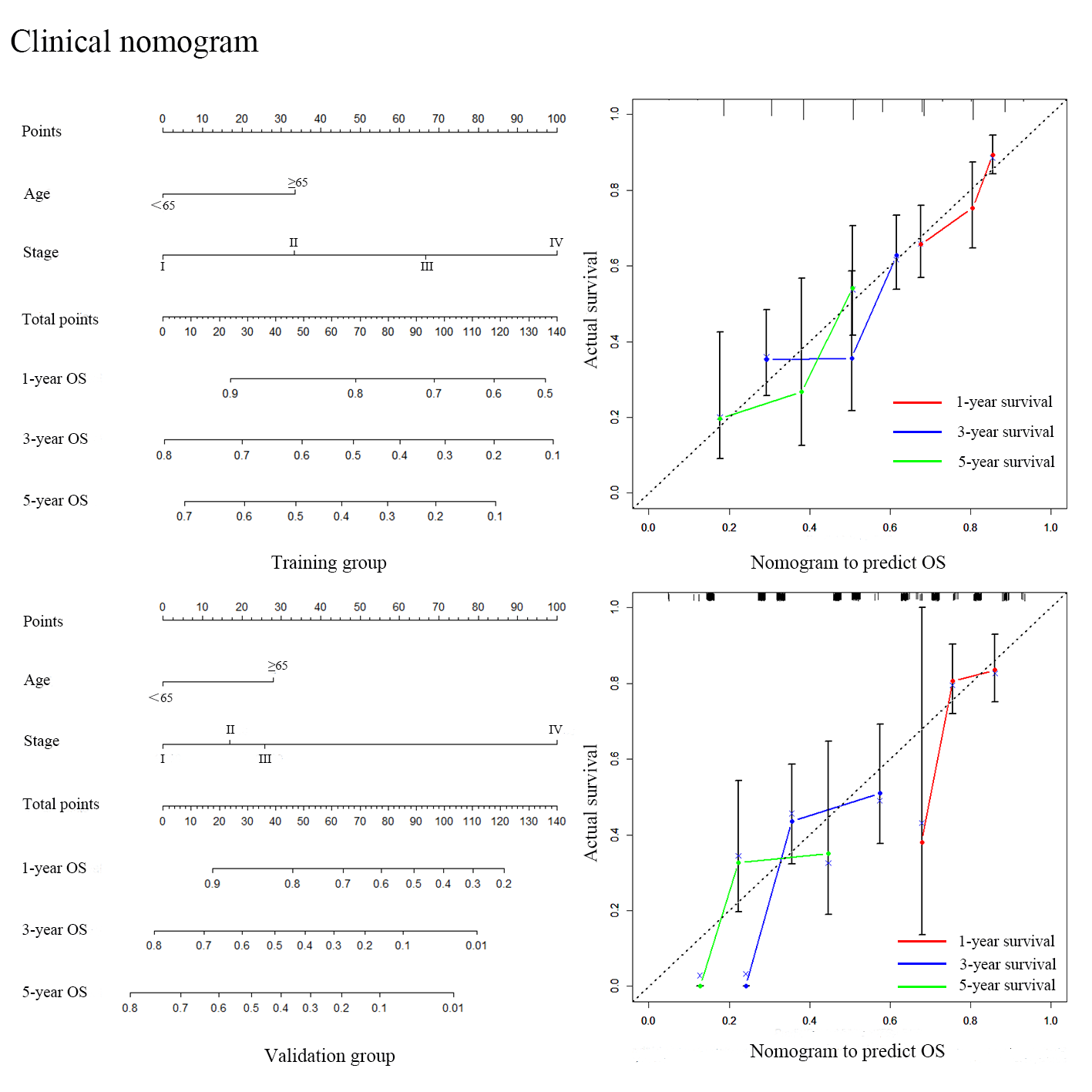


**Figure S11:** Clinical nomogram and its calibration plot in training group and validation group


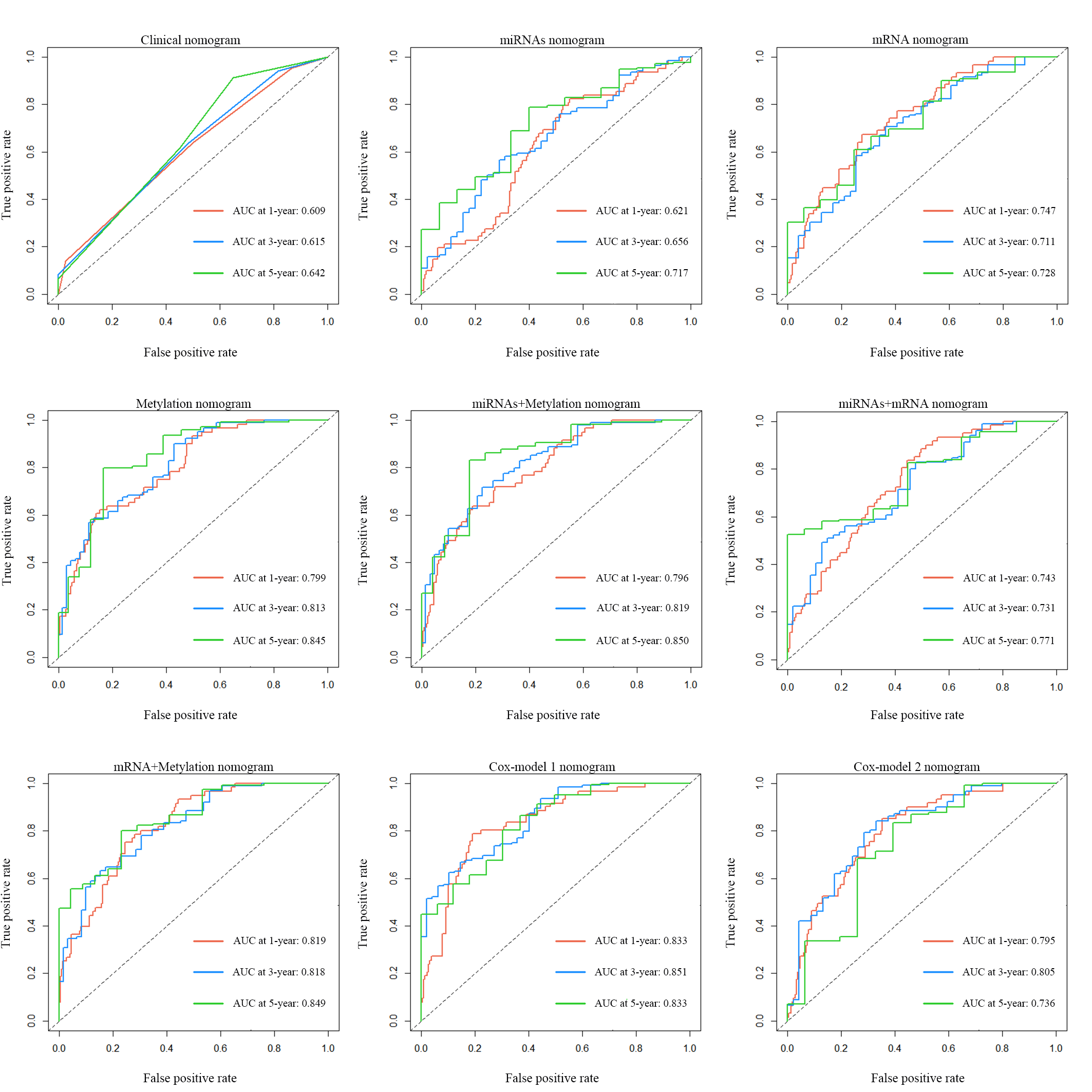


**Figure S12:** Time-dependent ROC curves on 1, 3 and 5-year for each nomogram in training group


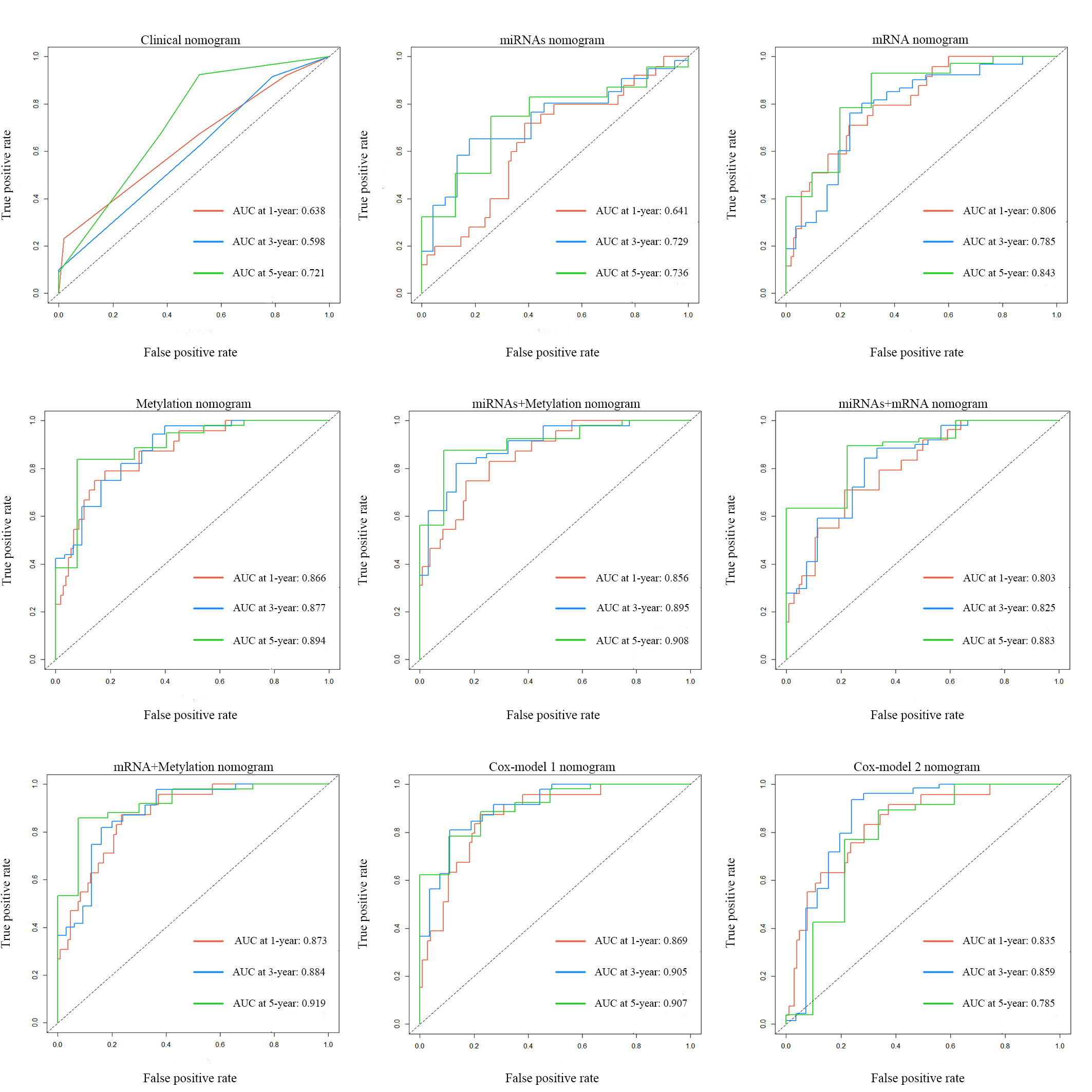


**Figure S13:** Time-dependent ROC curves on 1, 3 and 5-year for each nomogram in validation group


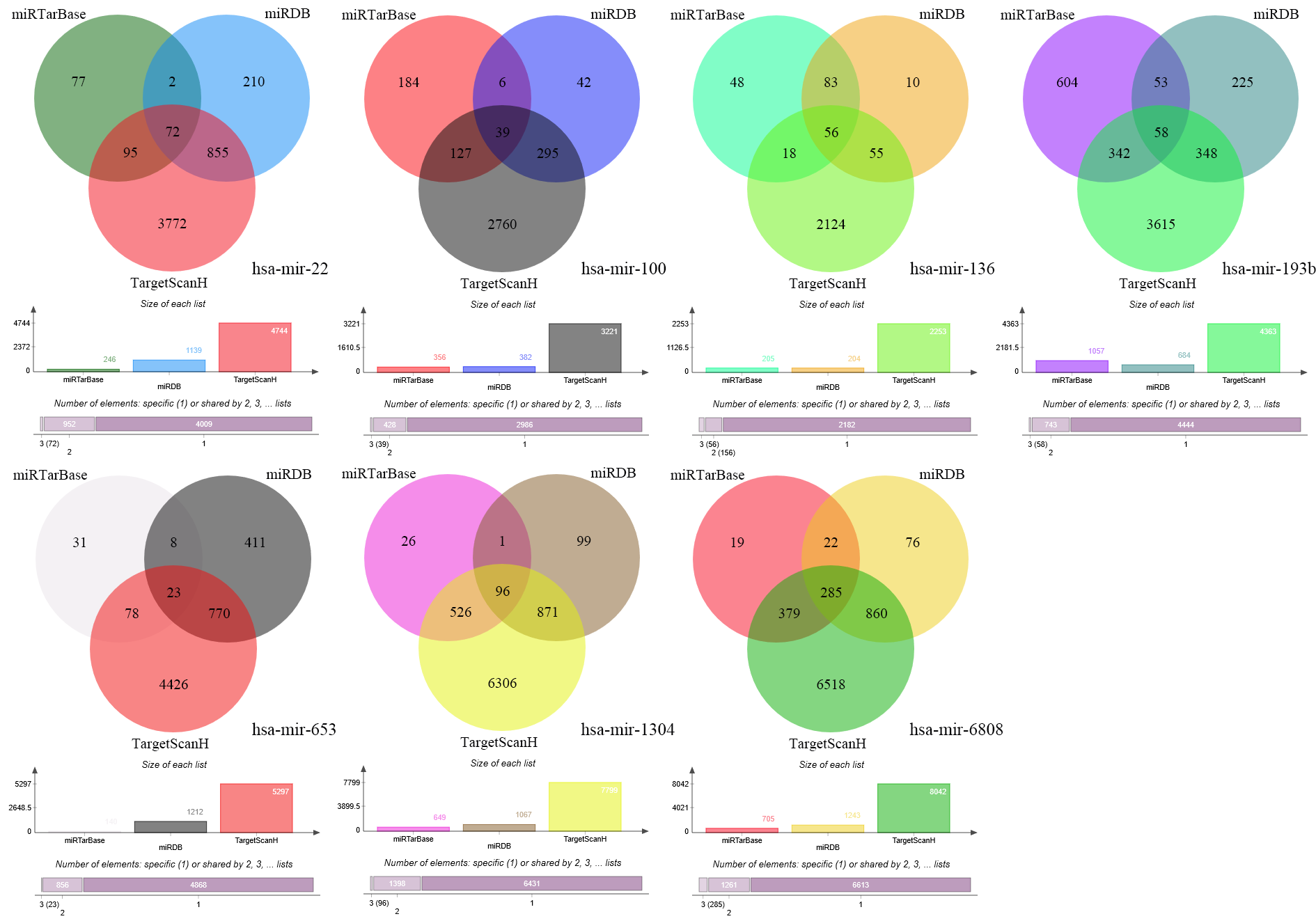


**Figure S14:** venn diagrams for the target genes of each miRNA


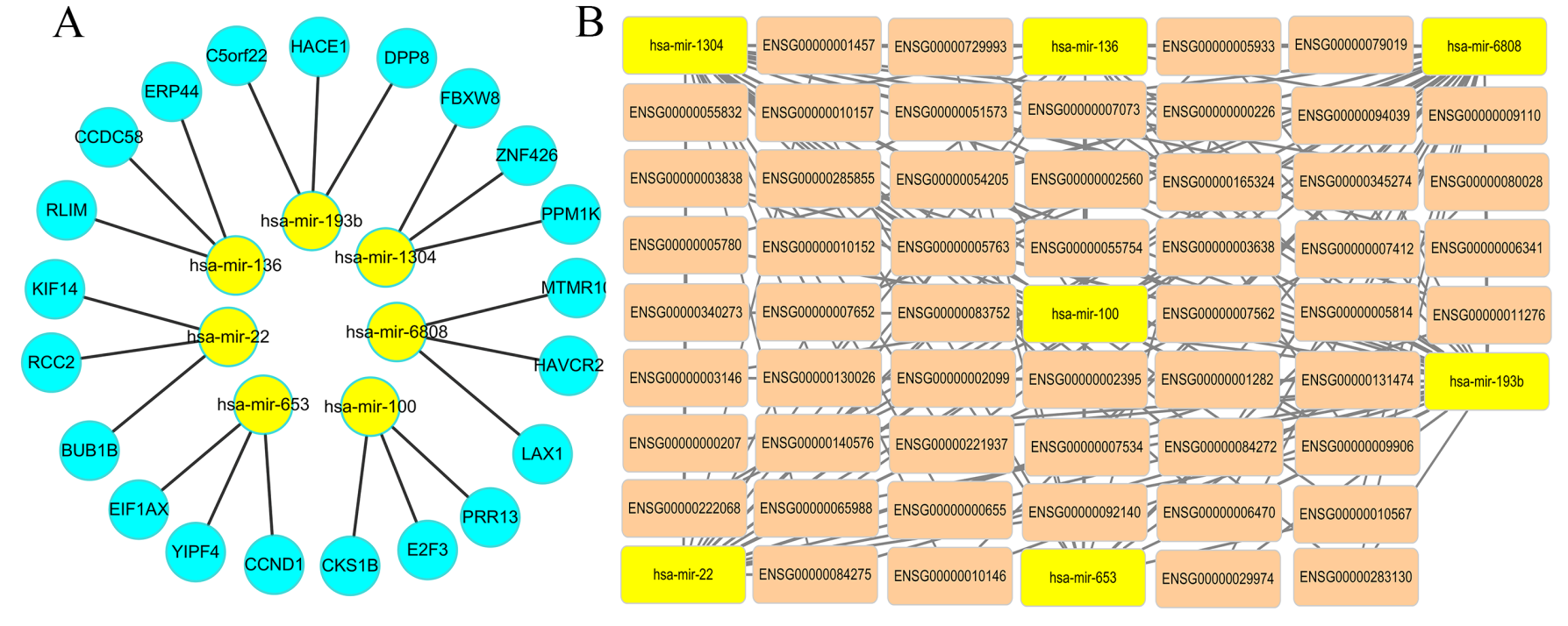


**Figure S15:** Three most potential target genes for each miRNA (A) and the miRNA-target genes co-expression network (B)


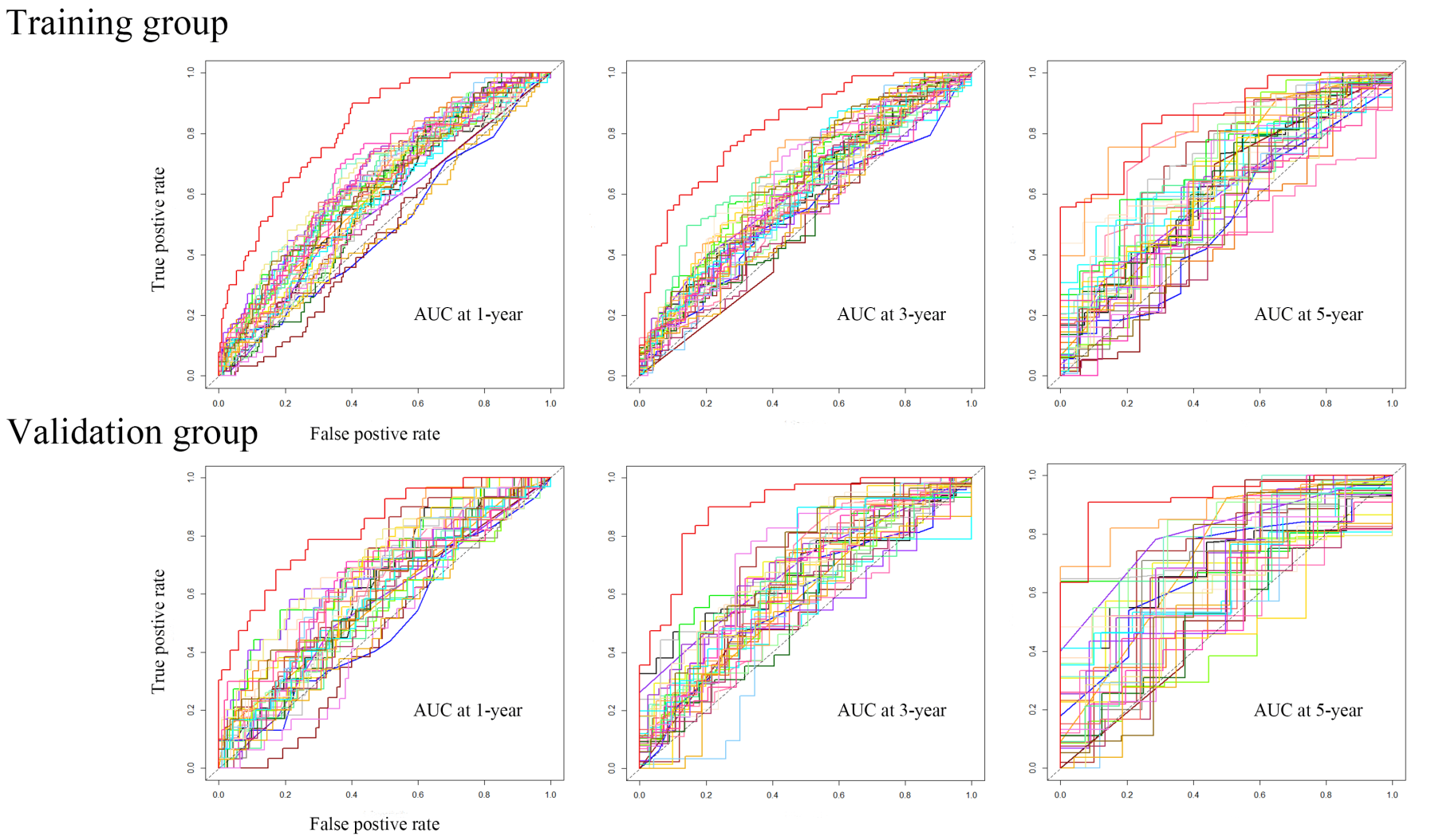


**Figure S16:** Time-dependent ROC curves on 1, 3 and 5-year for each signature in genome-wide network. Each color represents different signature: red2 (genomics nomogram), orange (clinical nomogram), yellow (hsa-mir-100), blue (hsa-mir-1304), black(hsa-mir-193b), brown (hsa-mir-22), gray (hsa-mir-136), palevioletred1 (hsa-mir-653), purple2 (hsa-mir-6808), green2 (NRP1|8829), darkred (SOX14|8403), darkorchid3 (CPNE8|144402), darkorange2 (MAGED1|9500), cyan (RNF144A|9781 ), cornsilk4 (ZNF22|7570), hotpink1 (DUSP1|1843), chartreuse (LOC91450|91450), aquamarine2 (cg02223323), darkgreen (cg00481239), maroon1 (cg07020967), palegreen (cg08859156) gold (cg12485556), purple1 (cg14791193), antiquewhite (cg15861578), khaki2 (cg15486740) seagreen2 (cg20100408), lightskyblue (cg20350671), maroon (cg22395807), indianred2 (cg24361571), tan1 (cg25361506), peachpuff (cg25622155), turquoise1 (cg25161386), orchid2 (cg22740006), darkgoldenrod2 (cg22813794), darkgoldenrod4 (cg26014401), violetred1 (cg26856948)


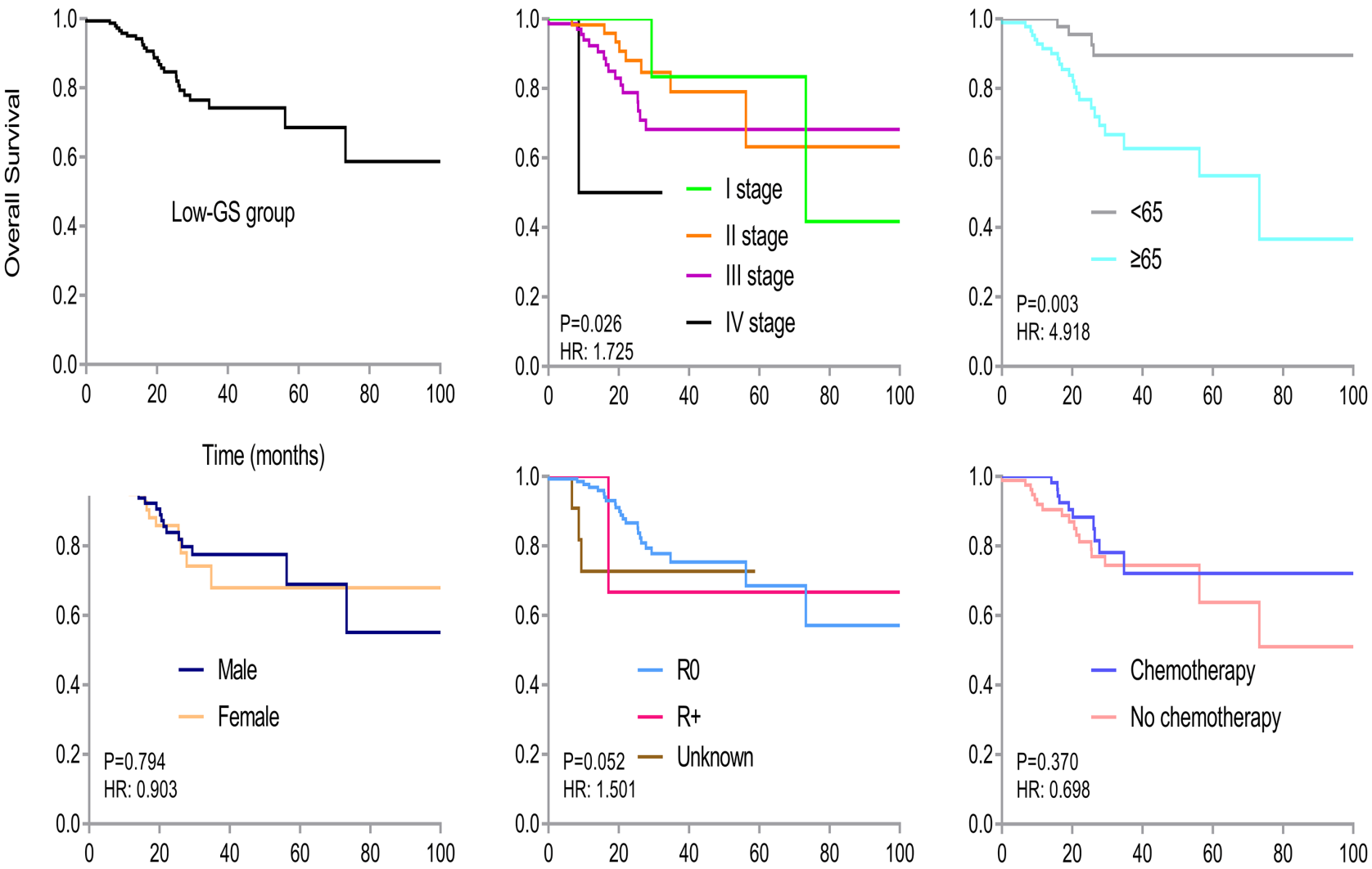


**Figure S17:** Kaplan-Meier curve of overall survival in low GS group stratified by clinical features (eg: stage)


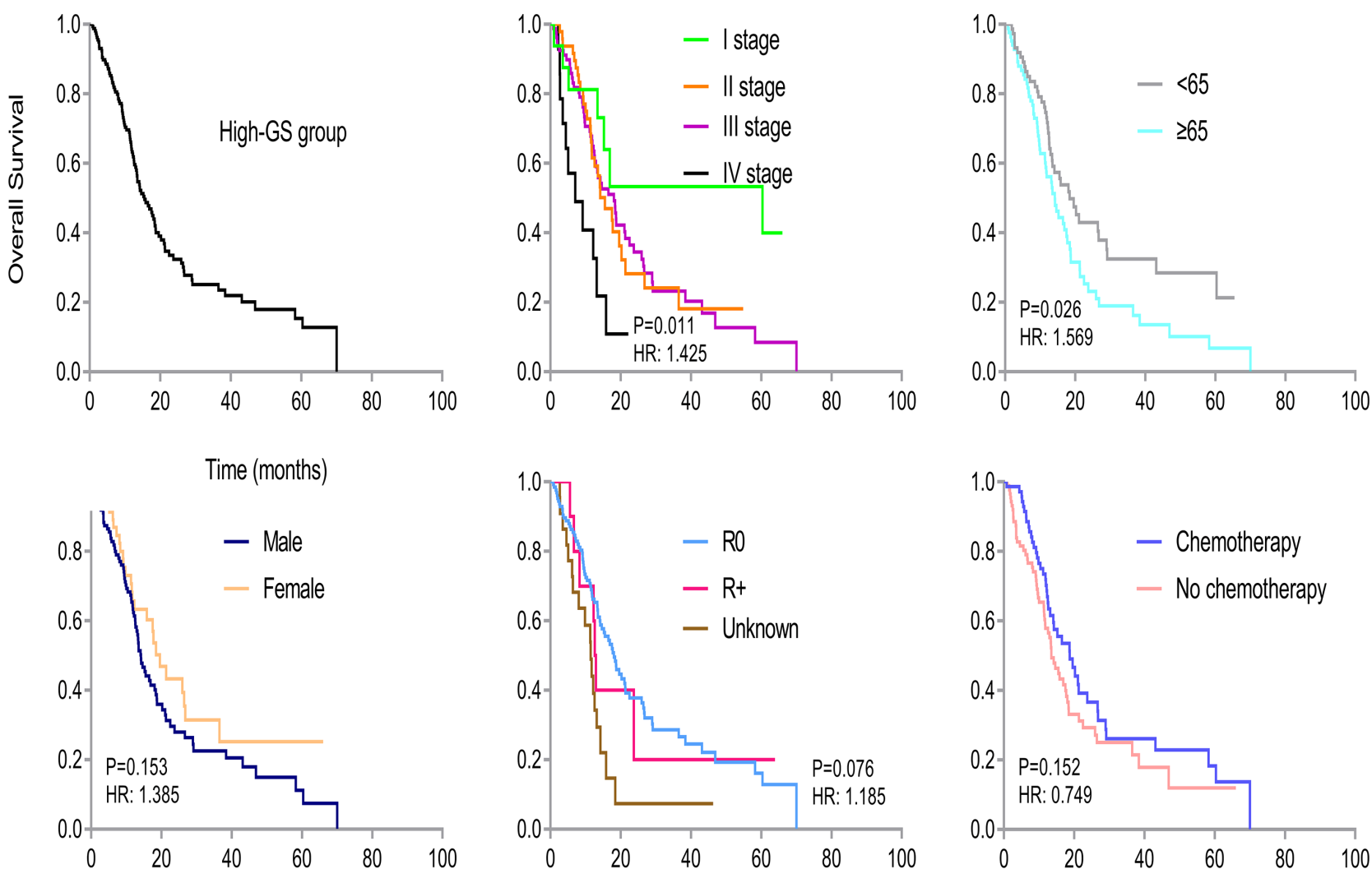


**Figure S18:** Kaplan-Meier curve of overall survival in high GS group stratified by clinical features (eg: stage)


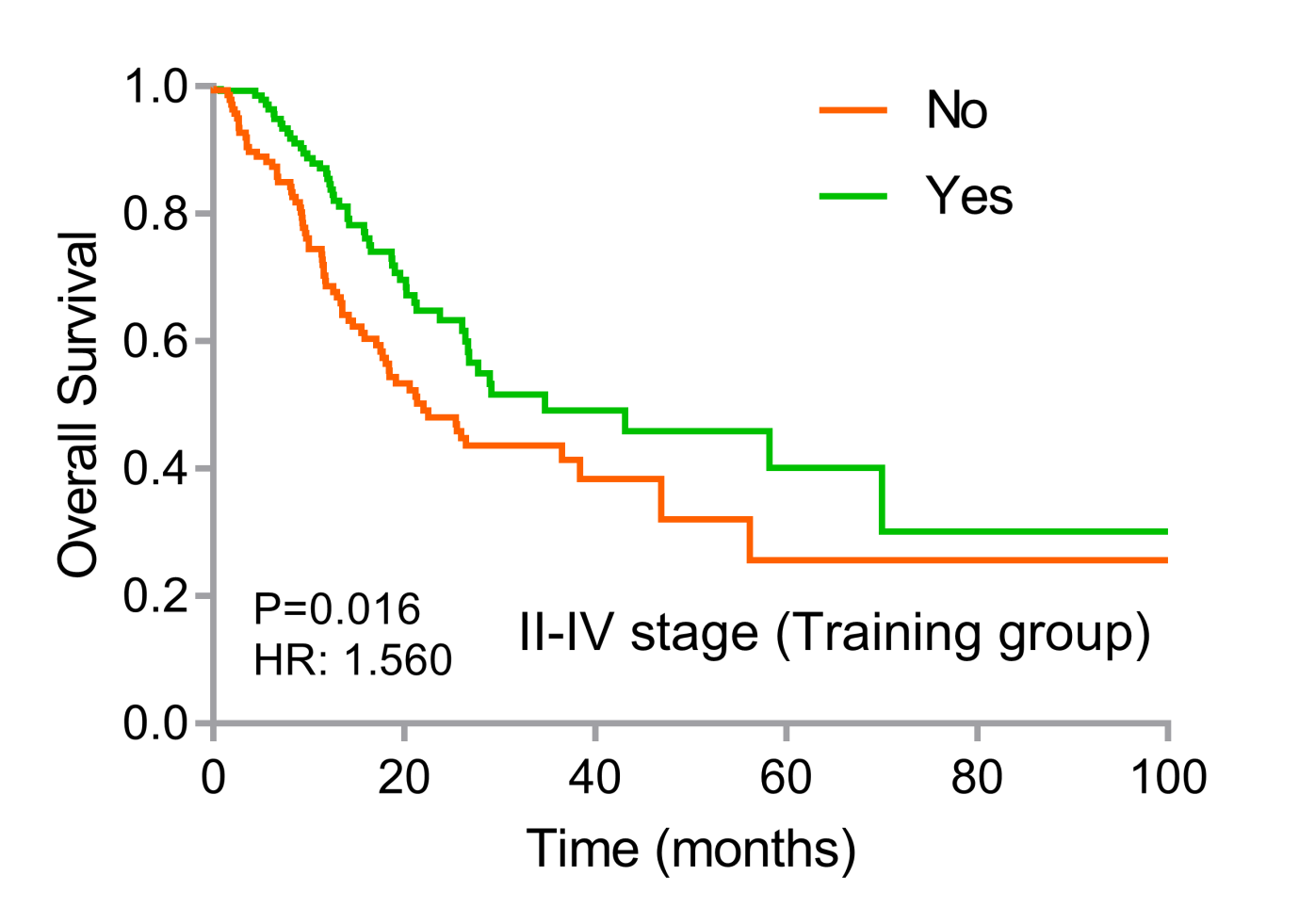


**Figure S19:** Kaplan-Meier curve of overall survival in II-III stage patients
